# Pipitzols and Isopipitzols, 1852 to 2026: A Review

**DOI:** 10.3390/molecules31183342

**Published:** 2026-09-20

**Authors:** Joel Martínez, Cristopher Williams Fuentes Cid, Adriana Lizbeth Rivera Espejel, María Inés Nicolás-Vázquez, María Z. Saavedra-Leos, Alberto A. Fajardo de la Rosa, René Gerardo Escobedo-González, René Miranda Ruvalcaba

**Affiliations:** 1 Departamento de Ciencias Químicas, Facultad de Estudios Superiores Cuautitlán Campo 1, Universidad Nacional Autónoma de México, Cuautitlán Izcalli 54740, Estado de México, Mexico; jomtz@comunidad.unam.mx (J.M.); 316048734@cuautitlan.unam.mx (C.W.F.C.); riveraespejeladriana@gmail.com (A.L.R.E.); 2 Coordinación Académica Región Altiplano, Universidad Autónoma de San Luis Potosí, Carretera Cedral km 5+600, Ejido San José de las Trojes Matehuala 78700, San Luis Potosí, Mexico; zenaida.saavedra@uaslp.mx; 3 Laboratorio de Ingeniería Química, Facultad de Química, Universidad Nacional Autónoma de México, Alcaldía Coyoacán, Ciudad de México 04510, Mexico; aafdlr@ciencias.unam.mx; 4 Department of Industrial Maintenance, Technological University of the City of Juárez, Av. Universidad Tecnológica No. 3051, Col. Lote Bravo II, City of Juárez 32315, Chihuahua, Mexico; rene_escobedo@utcj.edu.mx

**Keywords:** pipitzols, isopipitzols, timeline, quantum chemistry, computational chemistry, pharmacological activity

## Abstract

A thorough review of pipitzols and isopipitzols, covering the period 1852–2026, is presented. Perezone, the precursor of these compounds, is considered to be the first secondary metabolite isolated on the American continent, a phyto-molecule widely studied with regard to its spectra, chemical transformations, and especially its isolation and pharmacological activities. Perezone has several isomers: isoperezone, a synthetic molecule; and α- and β-pipitzols, two natural isomers of cedrenoid type achieved by thermal *exo* [4+2] cycloadditions. Isoperezone can be isomerized to β-isopipitzol through an *endo* [4+2] cycloaddition, while α-isopipitzol can be obtained from perezone by a [1+3] dipole addition. For this review, we conducted a comprehensive literature search, employing the main Scopus, SciFinder^®^, and ResearchGate databases, which identified 148 references. We found that the most recent report on α- and β-pipitzols appeared in 2024, on α-isopipitzol appeared on 2 September 2026, and on the four isomers was published on 29 January 2026.

## 1. Introduction

### 1.1. Synopsis

**Figure 1:**
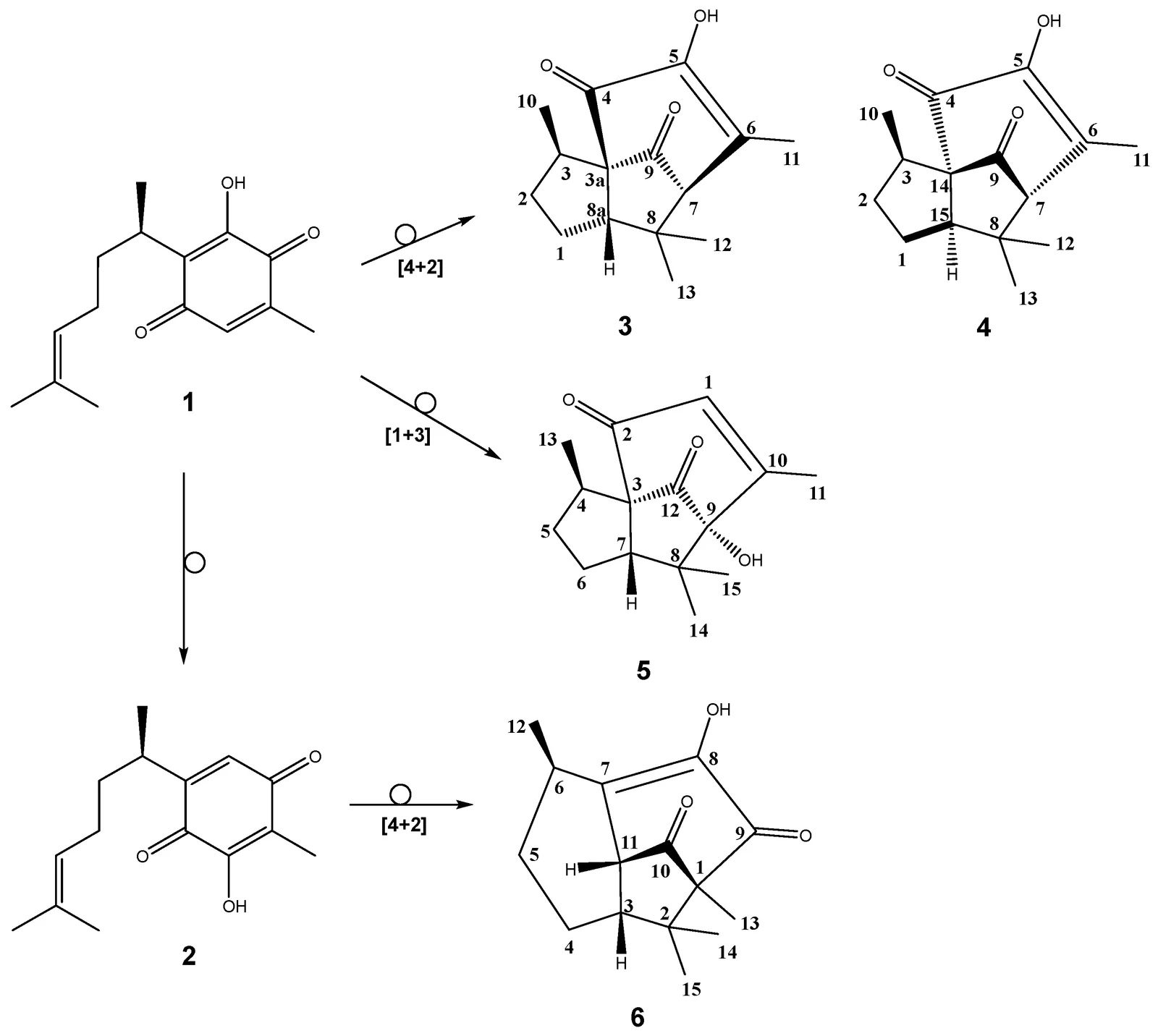
Structures of perezone (**1**), isoperezone (**2**), α-pipitzol (**3**), β-pipitzol (**4**), α-isopipitzol (**5**), and β-isopipitzol (**6**), and their numbering.

Perezone (**1**, Figure 1), first reported in 1852, is acknowledged to be the first secondary metabolite isolated on the American continent, and has been broadly studied with regard to its spectra, synthesis, isolation, and pharmacological properties [1,2]. It is important to note that, considering the target compounds in this review, perezone has several isomers, as exhibited in Figure 1. Isoperezone (**2**) is produced by the chemical transposition of the hydroxyl group in **1**. Furthermore, in various *Acourtia* plant specimens, perezone is frequently accompanied by two natural isomers of cedrenoid type, namely, α- and β-pipitzols (**3** and **4**, respectively) [3]. These are obtained via thermal *exo* [4+2] cycloadditions of **1**, reactions that proceed in the presence of diverse Lewis acids [4,5,6]. Isomer **2** can be isomerized to β-isopipitzol (**6**) by an *endo* [4+2] cycloaddition, while the α-isomer (**5**) can be obtained from perezone by a [1+3] dipole addition [4,5,6].

As part of our research program on natural products involving green chemistry, we have focused on the extraction, isolation, modification, and computational studies of perezone and several derivatives, engendering molecules with significant pharmacological activities [1,2,7]. The goal of this work is to provide a review of analogs **3**–**6**, covering the period 1852–2026, and to highlight that, to the best of our knowledge, this is the first review of **3**–**6**. Additionally, this review includes in silico and pharmacological studies of the title compounds conducted by our research group, presenting data that have not been reported elsewhere.

### 1.2. General Objective

The objective was to acquire important information from specialized databases (Scopus, SciFinder^®^, and ResearchGate) on α-pipitzol (**3**), β-pipitzol (**4**), α-isopipitzol (**5**), and β-isopipitzol (**6**) to generate a suitable manuscript covering publications related to the target products, incorporating fully updated information, and presenting the results in a convenient grouped format.

### 1.3. Chronological Study

1. An exhaustive search was carried out for scientific publications concerning the target compounds, known as pipitzols and isopipitzols, using the Scopus, SciFinder^®^, and ResearchGate databases.
2. The publications were grouped, considering isolation and structure, synthesis and stereoselectivity, computational studies, and pharmacological studies.
3. Salient data from each of the acquired manuscripts were summarized and structured for the review format.

## 2. Methods

The literature search was carried out using the main SciFinder^®^, Scopus, and ResearchGate databases, covering the period from perezone isolation (1852) [8] to 2 September 2026, considering the following keywords: perezone; pipitzols; isopipitzols; α-pipitzol; β-pipitzol; α-isopipitzol; β-isopipitzol.

## 3. Results and Discussion

Our exhaustive literature search yielded a relatively small number of references; these are summarized in Table 1, with important commentaries denoted a–k below.

(a) Only two studies concerning pharmacological activity were found, which focused on the anti-diabetic activity of the roots of *Acourtia thurberi* decoction, inhibitory activity against COX-2 and PARP-1 enzymes, and cytotoxic activity against five cancer cell lines.
(b) Only one research study involved mass spectrometric determinations of **1**–**4** and **6**.
(c) The most recent synthesis of **3** was published in 2026.
(d) Unequivocal assignments of the ^1^H NMR signals for **3** were reported in 2017.
(e) VCD studies on **3** and **4** were reported in 2022 and the spectra were compared with theoretical predictions.
(f) To explain the transformation from **1**, the transition states of **3** and **4** were reported in 2024.
(g) The synthesis and spectroscopic characterization of **6** were reported in 1994 and 1996, respectively.
(h) α-isopipitzol (**5**) was characterized by XRD studies in 2000, and by NMR data in 2026.
(i) Computational calculations on the target compounds (geometrical, electronic properties, and docking, among others) were reported by our research group in 2026.
(j) This review of the title molecules **3**–**6** is organized chronologically in the following sections: Section 3.1 Isolation and structure; Section 3.2 Synthesis and stereoselectivity; Section 3.3 Computational studies; Section 3.4 Pharmacological studies; Section 3.5 Complementary information; and Section 3.6 Future perspectives from our laboratory.
(k) It is very important to highlight that in some of the original papers, several inconsistencies exist (nomenclature, concepts, synthetic conditions, and substituents (R, R’, etc.), among others). As the authors of this review, we agreed to maintain the status of the original publication, providing a summary of any addenda and corrigenda at the end.

### 3.1. Isolation and Structure

#### 3.1.1. Fructicosina-Pipitzahuina by Severiano Pérez (1857)

Pérez documented a white crystalline substance as a component of pipitzahuac resin, initially named fructicosina and subsequently renamed pipitzahuina [9,10]. To the best of our knowledge, after our extensive literature search, this was the first information on pipitzols, but was lost over time. Pérez described the isolation following an accidental thermal transformation of perezone, and only some simple physical properties of this by-product were reported. Pérez judged that Río de la Loza’s perezone was a mixture of pipitzols and some other colored compounds.

#### 3.1.2. Perezone (1913)

Perezone is an orange crystalline compound isolated from the roots of pipitzahuac in Mexico. Upon heating to 210 °C, it generates pipitzol, a colorless, crystalline [11], optically active isomeric product that does not react with aniline, hydroxylamine, or phenylhydrazine. When pipitzol is treated with an ethereal solution of methyl magnesium iodide, it forms tetrahydropipitzol, and when treated with acetic anhydride it produces acetylpipitzol; hydrolysis in an acid medium regenerates pipitzol. Treatment with aqueous sodium hydroxide solution yields an oil. One of the three oxygen atoms of pipitzol is phenolic; hence, a monoacetylated derivative is produced. This compound is not quinoid; it is colorless, and it is not a ketone since it does not react with hydroxylamine or phenylhydrazine.

#### 3.1.3. On the Constitution of Perezone (1935)

In 1906, the isolation of a colorless perezone derivative was reported, apparently with the same molecular formula as **1**. Further, in 1913, several derivatives were prepared, establishing empirical formulas for **3** and **4**. Notably, pipitzol was reported as being optically active, in contrast to perezone [12]. Comparing the melting points of **1** and its derivatives verified that they were different. Moreover, in testing for phenolic compounds with FeCl_3_ and for unsaturation with Br_2_, pipitzol gave a positive result in both cases. For the preparation of pipitzol, perezone was initially heated at 200 °C and subsequently at 300 °C, producing colorless crystals accompanied by a brown-colored oily residue. After recrystallizing from light petroleum ether, a product with mp 141 °C was obtained.

#### 3.1.4. A Correction to the Structure of Perezone (1965)

**Figure 2:**
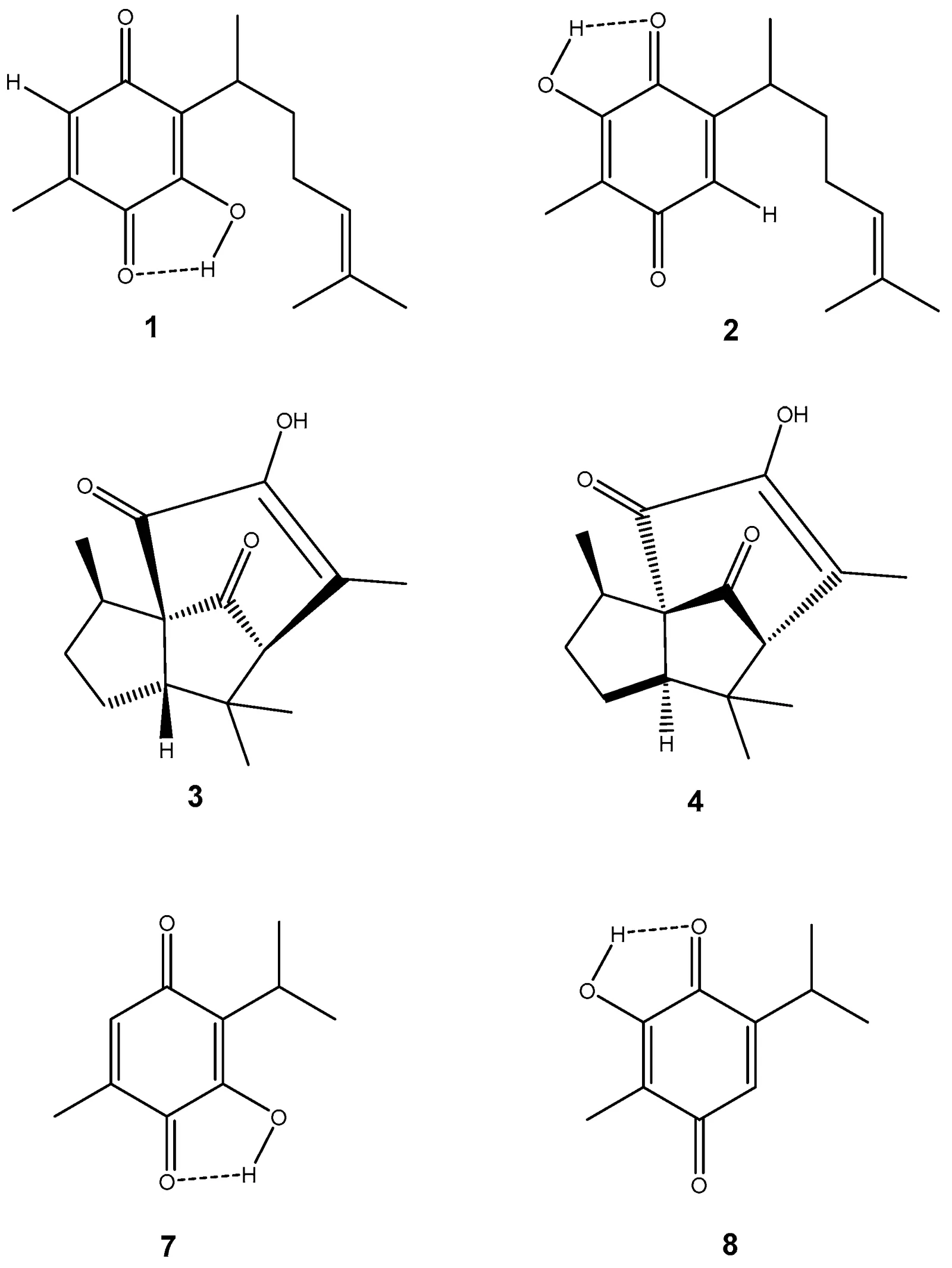
Corrected perezone (**1**), **2**-initial proposal, α- and β-pipitzols (**3** and **4**, respectively), 6-hydroxythymoquinone (**7**), and 3-hydroxythymoquinone (**8**). Adapted from Ref. [13].

Through an NMR study [13,14], the structure of perezone (**1** instead of **2** [12]) was corrected. It was found that the methyl group and the ring proton signals indicate that they are in an *ortho* relationship (Figure 2). Further evidence was provided by infrared spectrophotometry, with the relative positions of various bands correlating more closely with **7** than **8**. Structure **1** offers a more direct route to pipitzols than **2**, which involves cyclization via Michael addition of the double bond in the side chain with the quinone moiety, followed by a proton transfer to develop the tricyclic system.

#### 3.1.5. Terpenoids of *Perezia hebeclada* (1972)

**Figure 3:**
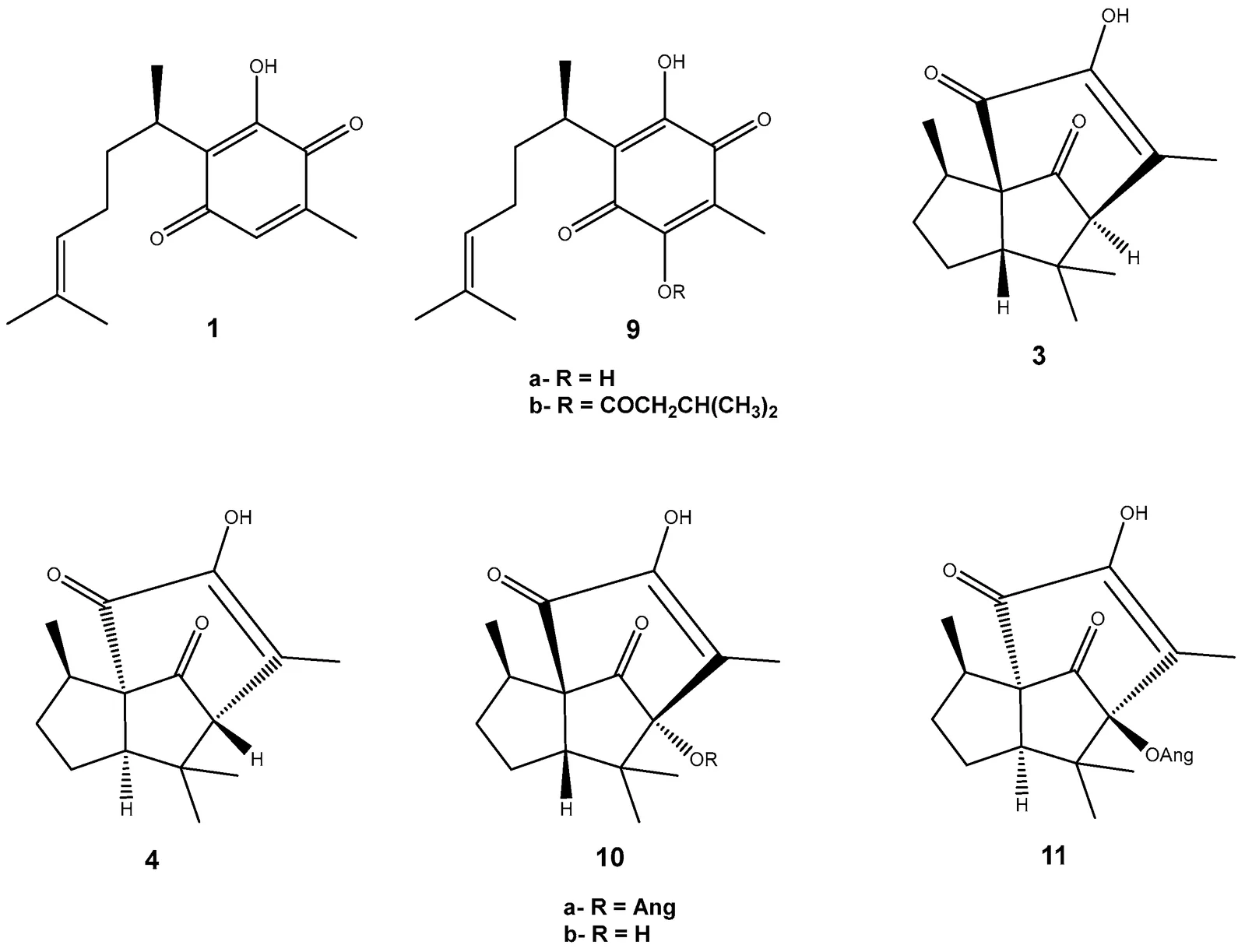
Structures of **1**, **3**, **4**, hydroxyperezone (**9a**), hydroxyperezone monoisovalerate (**9b**), α-perezol (**10a**), β-perezol (**11**), and γ-perezol (**10b**). Adapted from Ref. [15].

In a study of *Perezia hebeclada* roots [15], **1**, hydroxyperezone (**9a**), hydroxyperezone monoisovalerate (**9b**), and **3** and **4** were obtained, in addition to a colorless crystalline mixture from which, after fractional crystallization, another three new molecules were isolated: α-perezol (**10a**), β-perezol (**11**), and γ-perezol (**10b**) (Figure 3). The corresponding structures were established by correlating their spectrophotometric properties (UV and IR) and their ^1^H NMR data. For example, pipitzol and perezol displayed simple signals at 2.83 and 3.01 ppm, respectively. The corresponding stereochemistries were obtained by comparing the ORD curves. It should be noted that the authors suspected the existence of an additional compound with γ-perezol connectivity and the stereochemistry of β-perezol (**19**), although they could not isolate it.

#### 3.1.6. ^13^C-NMR Studies of Cedrenolides (1986)

**Figure 4:**
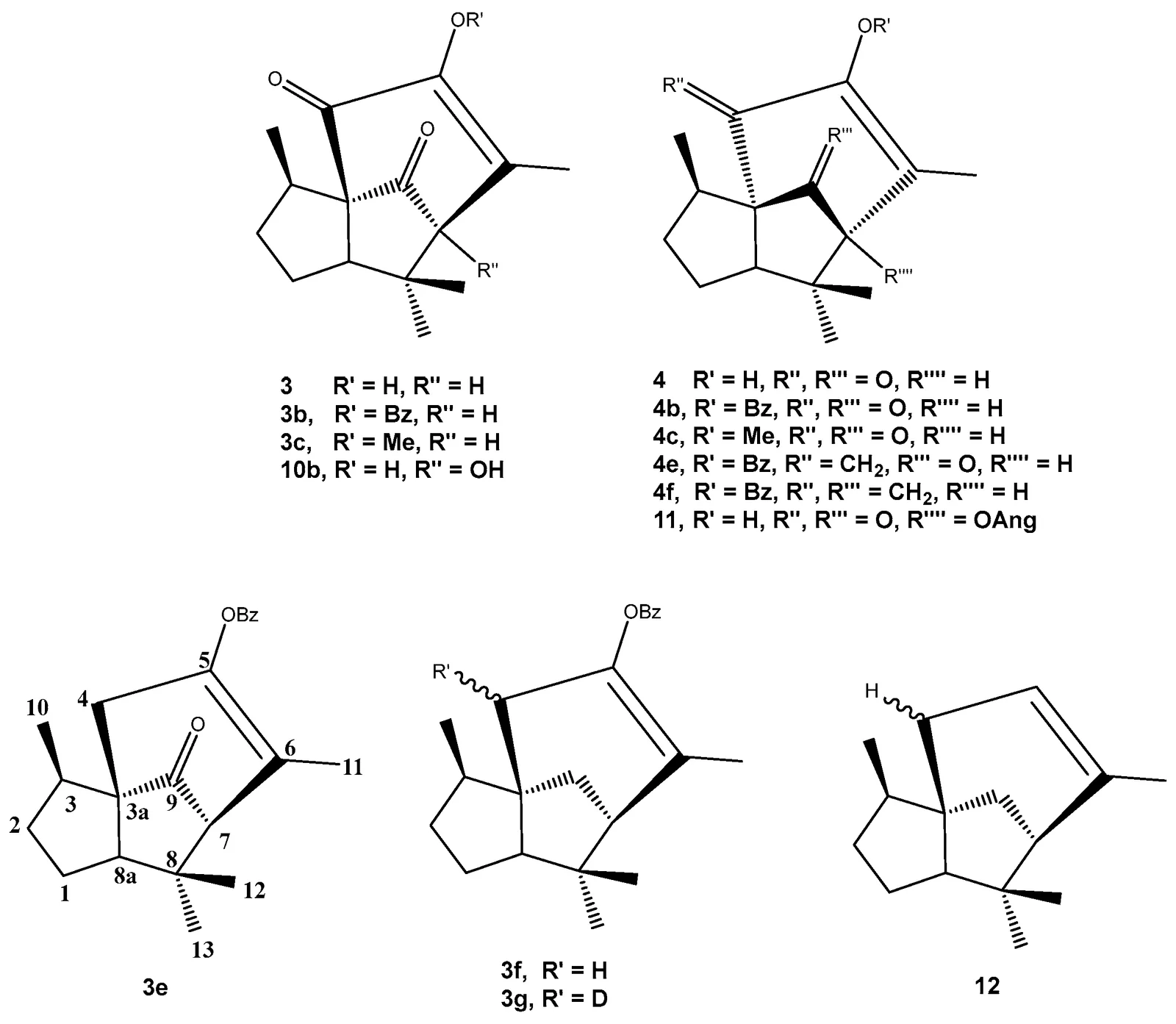
Derivatives of **3** and **4**, benzoates of 4-deoxo-α-pipitzol (**3e**), 4,9-*bis*-deoxo-α-pipitzol (**3f**), and 4-deutero-4,9-*bis*-deoxo-α-pipitzol (**3g**), and α-cedrene. Adapted from Ref. [16].

Cedrenolides belong to a group of sesquiterpenes. It is noteworthy that some of their members, such as cedrol, cedrene, α- and β-pipitzols, and many other derivatives, have not received sufficient systematic analysis of their ^13^C NMR data, which would be very useful for characterizing other molecules with similar structures. Although it is difficult to assign each signal due to rigidity and steric factors, some derivatives can be deuterium-labeled in key positions, permitting their distinction [16]. however, several important observations can be made regarding the target molecules (Figure 4).

In CDCl_3_/TMS as a solvent, the ^13^C NMR signals of C4 and C12 appeared at 193.7 and 24.4 ppm, respectively. These positions were then deuterium-labeled in α-, β-, and γ-perezols (**10a**, **11**, and **10b**, respectively). Consequently, comparing the spectrum of γ-perezol (**10b**) with that of α-pipitzol (**3**), the chemical shift for C7 (69 ppm) appeared at a higher field. Removing the hydroxyl group affected the methyl groups of C11, C12, and C13, shifting their signals to lower fields at 17.8, 24.4, and 25.5 ppm, respectively, whereas the signal of C10 at 14.9 ppm remained constant. Comparing the spectrum of **3** with that of α-pipitzol benzoate (**3e**), no significant variation in the chemical shifts was observed; the removal of the carbonyl group from C4 confirmed the assignments of these groups. With respect to *O*-methyl ether (**3c**), a difference was detected in terms of a shift to a lower field induced in C6 (141.8 ppm), corresponding to an effect induced by a change in the β-carbon of hydroxybenzoquinones.

Another important comparison can be made between the signals of C3a and C7 in 4-deoxo-α-pipitzol benzoate (**3e**) and 4,9-*bis*-deoxo-α-pipitzol benzoate (**3f**), which appear at a higher field at 60.2 and 63.2 ppm, respectively. A considerable amount of important information has been gleaned from the spectra of **4** and some of its derivatives (**4** to **4f**).

#### 3.1.7. Structure of a Mixed Crystal of Sesquiterpene Stereoisomers of 2,2,6,10-Tetramethyl-8,11-dioxotricyclo[5.3.1.0^3,7^]undec-9-en-9-yl Acetate (α- and β-Pipitzol Acetates) (1986)

Following the synthesis of α-pipitzol benzoate [17] and β-pipitzol acetate [18], the crystal structures of these stereoisomers were determined; the resulting data [19] allowed an important correlation to be made between their molecular geometries and those acquired previously.

**Figure 5:**
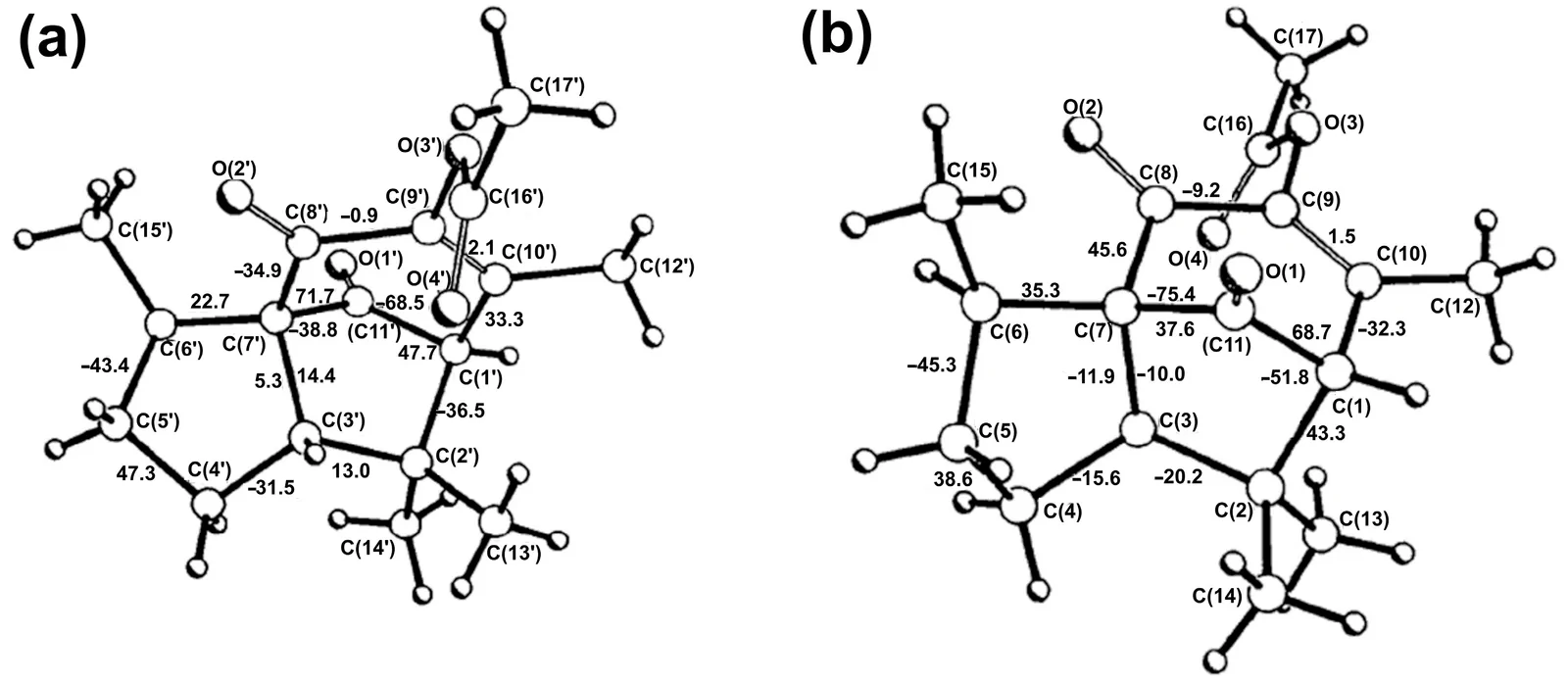
Molecular structures of (**a**) α-pipitzol acetate and (**b**) β-pipitzol acetate determined via X-ray diffraction. Adapted from Ref. [19].

Both compounds have two five-member rings (A and B) along with a six-membered ring (C), which are *cis*-fused. The α-pipitzol acetate adopts a conformation of envelope and half-chair, while the β-pipitzol acetate adopts a conformation that is half-chair and intermediate between half-chair and β-envelope (Figure 5). In both cases, the C ring has an out-of-plane 1,2-planar conformation.

**Figure 6:**
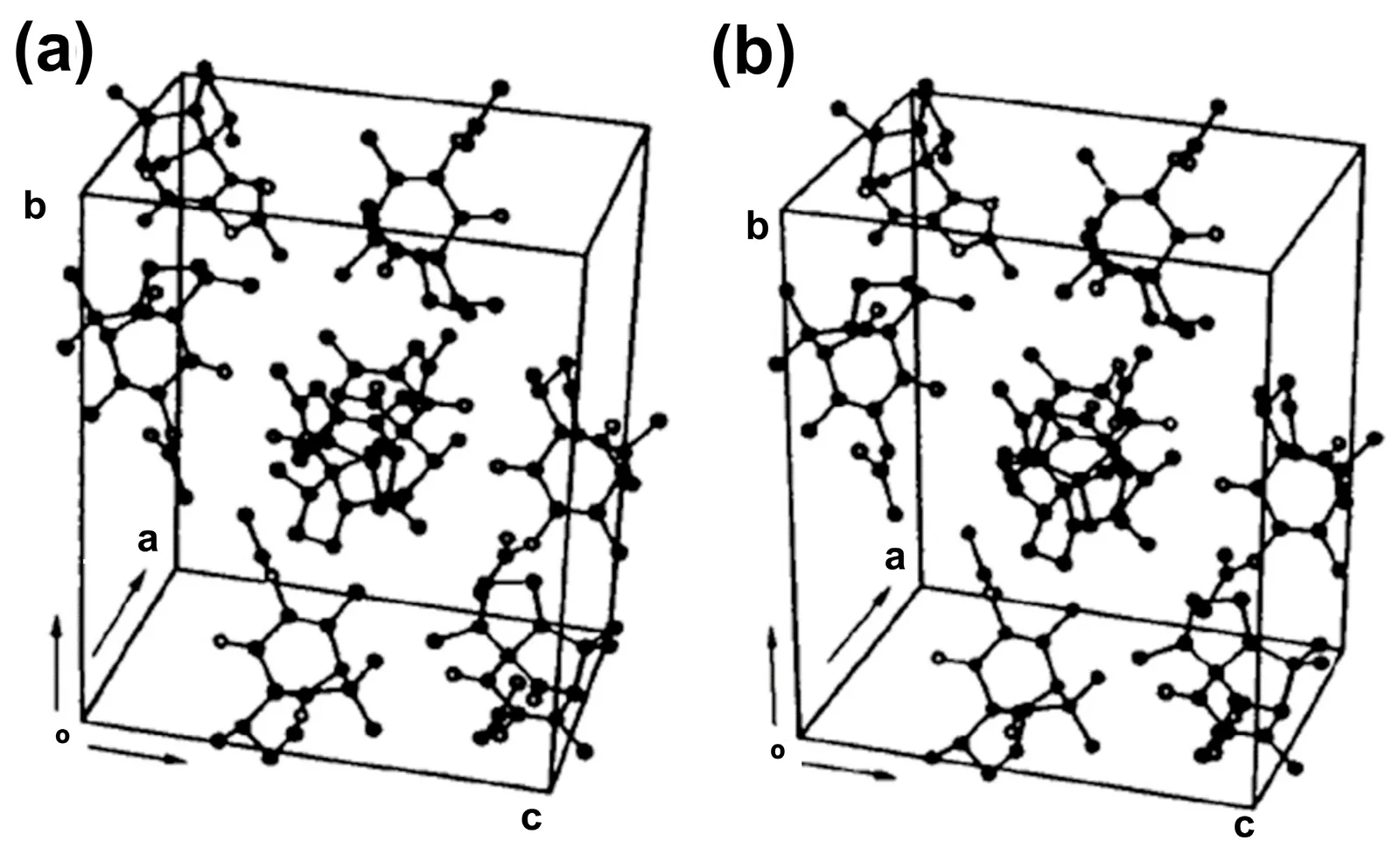
Crystal packing in the unit cells of (**a**) α- and (**b**) β-acetates. Adapted from Ref. [19].

The acetoxyl groups are oriented to minimize transannular repulsions between the O atoms and the C ring. They do not represent suitable hydrogen donors for the formation of hydrogen bridges. The molecules are held in the crystal by van der Waals forces (Figure 6). Finally, the molecular formulas of these compounds were corroborated by correlating them with that of (+)-cedrol.

#### 3.1.8. Structure of a Mixed Crystal of α- and β-Pipitzol (1:1), C_15_H_20_O_3_.C_15_H_20_O_3_ (1994)

Pipitzols are generated by the intramolecular addition of the double bond of the perezone (**1**) side chain to either side of the quinone moiety [20], resulting in the formation of three chiral centers. Therefore, α- and β-pipitzols (**3** and **4**, respectively) are enantiomeric at three centers, but have the same configuration (*R*) at C3.

**Figure 7:**
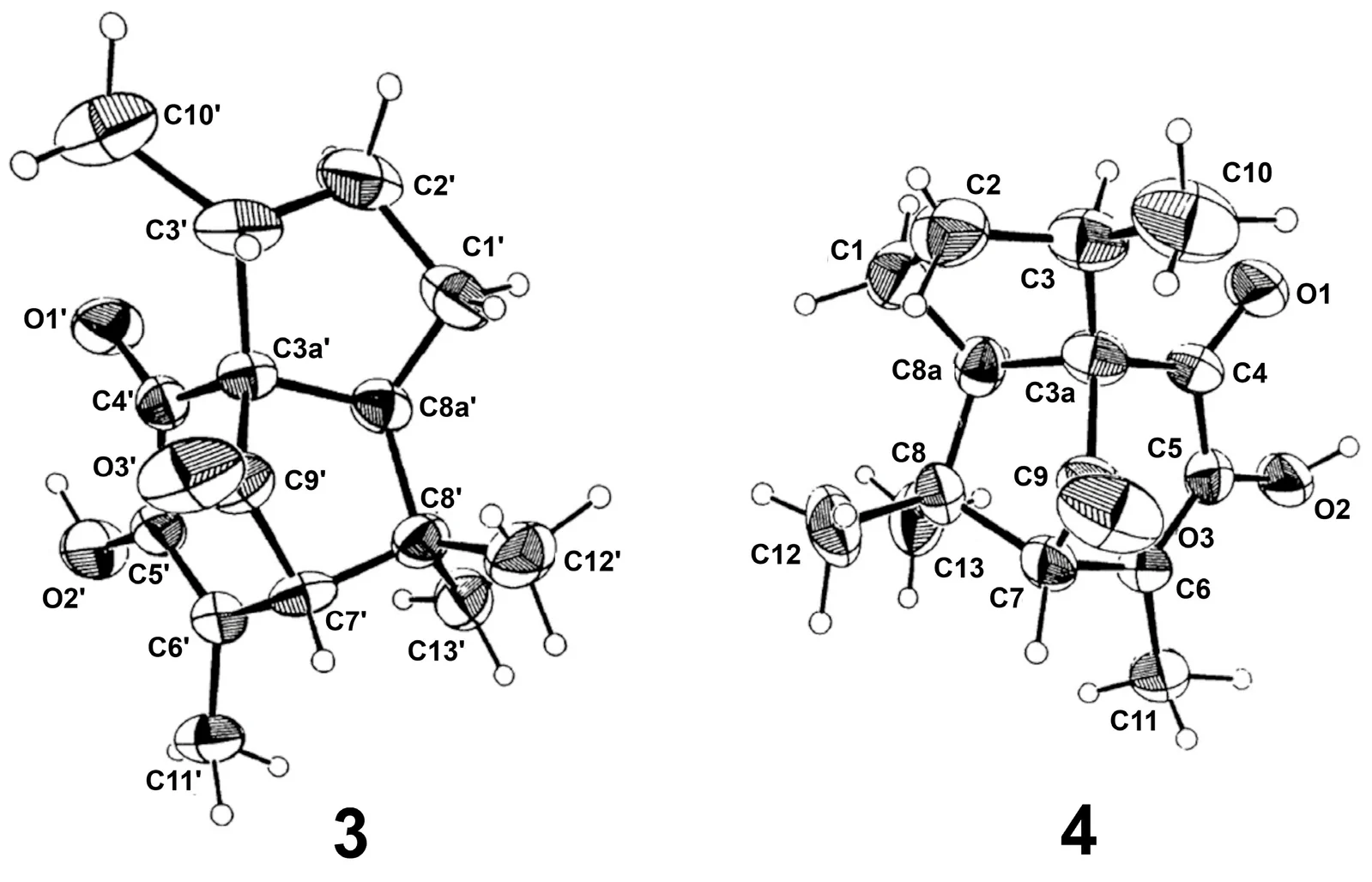
Views of **3** and **4** with 50% thermal ellipsoids. Adapted from Ref. [20].

This study presents the crystal structures of the pipitzol acetates (1:1 mixture of α and β). The unit cells each contain one pipitzol molecule and are related by a pseudo center of symmetry. The five-membered rings bearing the methyl substituent at C3 have approximate half-chair conformations, albeit with C1’ and C2’ more displaced from the plane in α-pipitzol (**3**), whereas in the case of β-pipitzol (**4**), C2 and C3 are more displaced. These conformations likely result from the reduction in intramolecular steric repulsions between the methyl groups (C10) and neighboring O atoms in the respective pipitzols (Figure 7).

The compounds are linked as α/β pairs through pseudo centers of symmetry by hydrogen bonds. The nonlinearity of the bonds could be due to cleavage by weak hydrogen bonds.

#### 3.1.9. The Structure of β-Isopipitzol (1994)

The isomerization of perezone (**1**) into isoperezone (**2**) through 1,2-carbonyl transposition catalyzed by 3,4,5,6-tetrahydro-2-pyrimidinethiol has recently been reported. When **2** is heated to its boiling point for 30 min or refluxed in toluene or xylene for several hours, it remains unchanged [4]; however, when **2** is treated with two equivalents of ZnBr_2_ in CH_2_Cl_2_ at room temperature for 72 h, three products are obtained—namely, perezinone, dihydroperezone and β-piptzol (**6**)—as a colorless tricyclic compound in a 20% yield.

The structure of **6** was established using chemical and spectroscopic methods. This compound tests positive for FeCl_3_, exhibits UV absorptions at 244 (sh) and 284 (nm), and its IR spectrum features bands at 3464 (OH), 1756 (cyclopentanone), and 1676 and 1632 cm^−1^ (six-membered enolized α-diketone). In EI-MS analysis, it exhibits a molecular ion at *m*/*z* 248 and a base peak at *m*/*z* 167. The ^1^H NMR spectrum of **6** features three singlet and one doublet methyl signals, an intense singlet at 5.98 ppm, a multiplet due to a single proton at 3.17 ppm, and a doublet at 3.12 ppm, as well as four multiplet signals due to one proton. In the ^13^C NMR spectrum, signals due to non-protonated carbon atoms appear between 140 and 207 ppm, along with four CH_3_, two CH_2_, three CH, and two quaternary carbons signals. Moreover, the stereochemistry was supported by NOEDS data, and the signals of Me-12, H-11, and H-3 indicated that they are in a *cis* relationship.

Regarding the formation of α- and β-pipitzols (**3** and **4**, respectively), it is mechanistically appropriate to suggest that it occurs through an *exo*-transition state, which is preferred in Diels–Alder intramolecular cycloadditions when three atoms are involved. However, in the case of β-isopipitzol (**6**), the *endo* transition state is not less impeded, and this is supported by a coupling constant of J = 7.2 Hz between H-3 and H-11, consistent with a dihedral angle of 33.2° (molecular mechanics program MM2) in the *endo* adduct.

**Figure 8:**
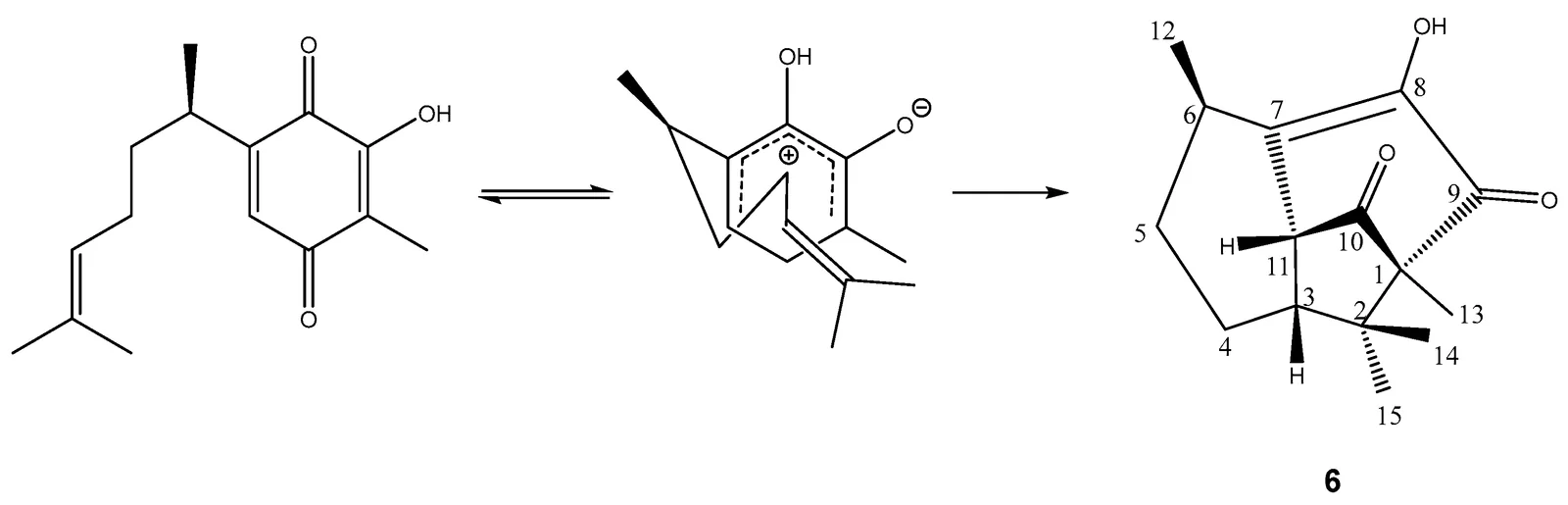
Proposal for a mechanism for the transformation of **2** to β-isopipitzol (**6**). Adapted from Ref. [4].

Finally, it should be noted that the transformation of **2** to **6** represents a rare example of an intramolecular [4+2] cycloaddition via a cyclopentadienyl, 2-substituted cation (Figure 8).

#### 3.1.10. β-Isopipitzol (1996)

In a report concerning the conversion of **2** to **6** catalyzed by ZnBr_2_, it was proposed that this transformation proceeds through an intramolecular *endo* [4+2] cycloaddition, based on the conformation and molecular geometry established by X-ray crystallography [5]. Molecule **6** is composed of three rings, two six-membered rings (A and C), and one five-membered ring (B). The A ring has a boat conformation with the methyl group at C-12 oriented equatorially; rings A and B are *cis*-fused at C3-C11, with H3 and H11 in a β-*cis* orientation, while rings B and C are fused at C1-C10-C11. Ring C has a distorted 10β couch conformation. Rings A and C are fused at C7-C11; the enol moiety O8-C8-C7 forms an angle of 105.1(3)° with C7-C6-C12 of ring A, and C1-C2-C3-C11 of ring B forms an angle of 131.8(3)° with C1-C10-C11. This ring has an approximate half-chair conformation, as seen in α- and β-pipitzols (**3** and **4**, respectively).

**Figure 9:**
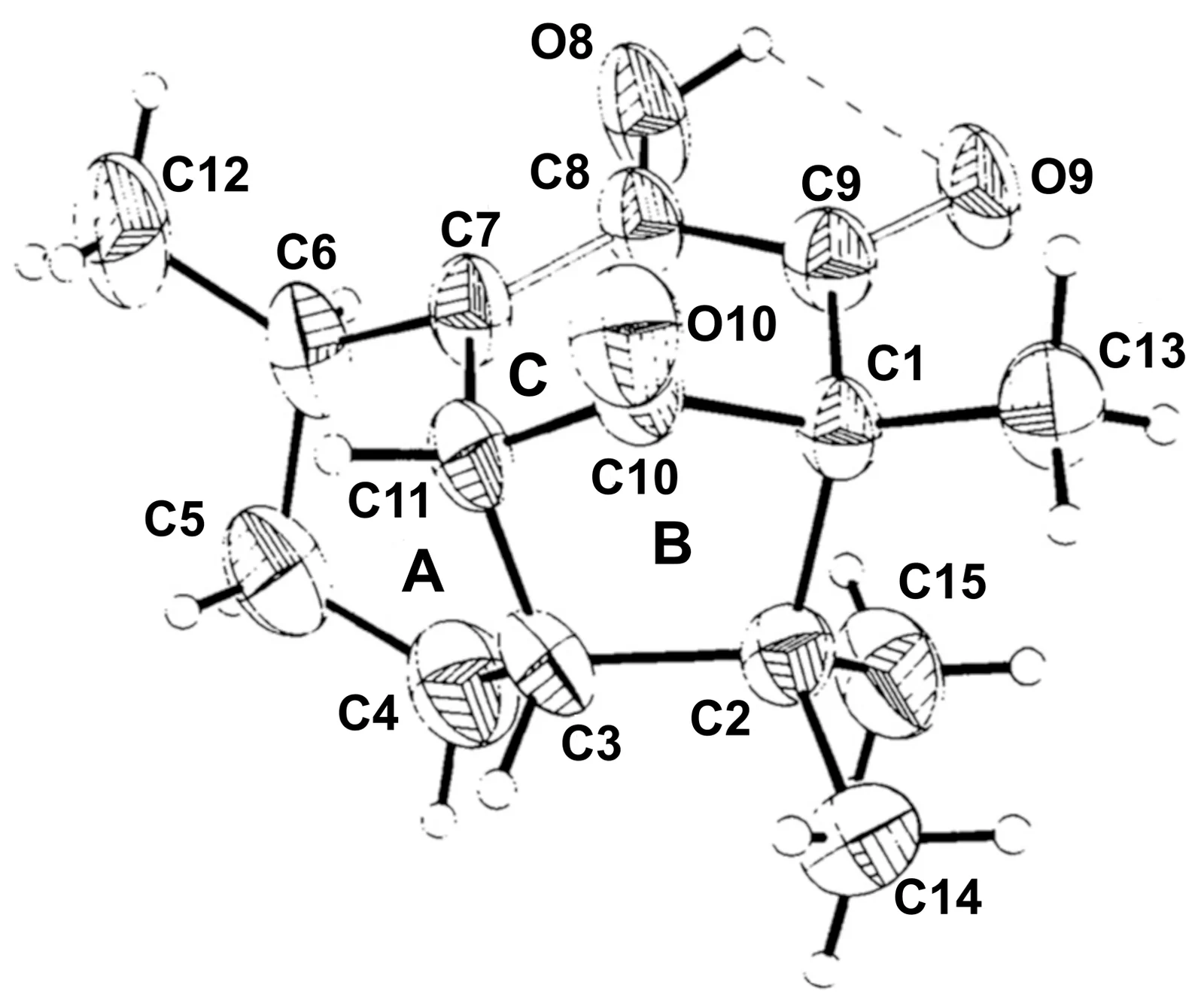
Molecular structure of β-isopipitzol (**6**). Adapted from Ref. [5].

The structural determination of β-isopipitzol (**6**) established it as (1*R*,3*R*,6*R*,11*R*)-8-hydroxy-1,2,2,6-tetramethyltricyclo[5.2.2.0^3,11^]undec-7-ene-9,10-dione, with the molecular formula C_15_H_20_O_3_. It should be noted that the absolute configurations, C3 (*R*) and C11 (*R*), were correctly assigned, having established the configuration at C6 (which bears a methyl group) based on single-crystal X-ray diffraction analysis (Figure 9). These assignments were consistent with previously published spectroscopic data.

In summary, **6** adopts an enol form with a double bond at C7=C8 and O8-H8 as the hydroxyl group. There are two notable hydrogen bonds, intramolecular O8-H8⋯O9 and intermolecular O8-H8⋯O10, which stabilize the crystal structure.

#### 3.1.11. The Mass Spectral Fragmentation of Perezone and Related Compounds (1997)

**Scheme 1:**
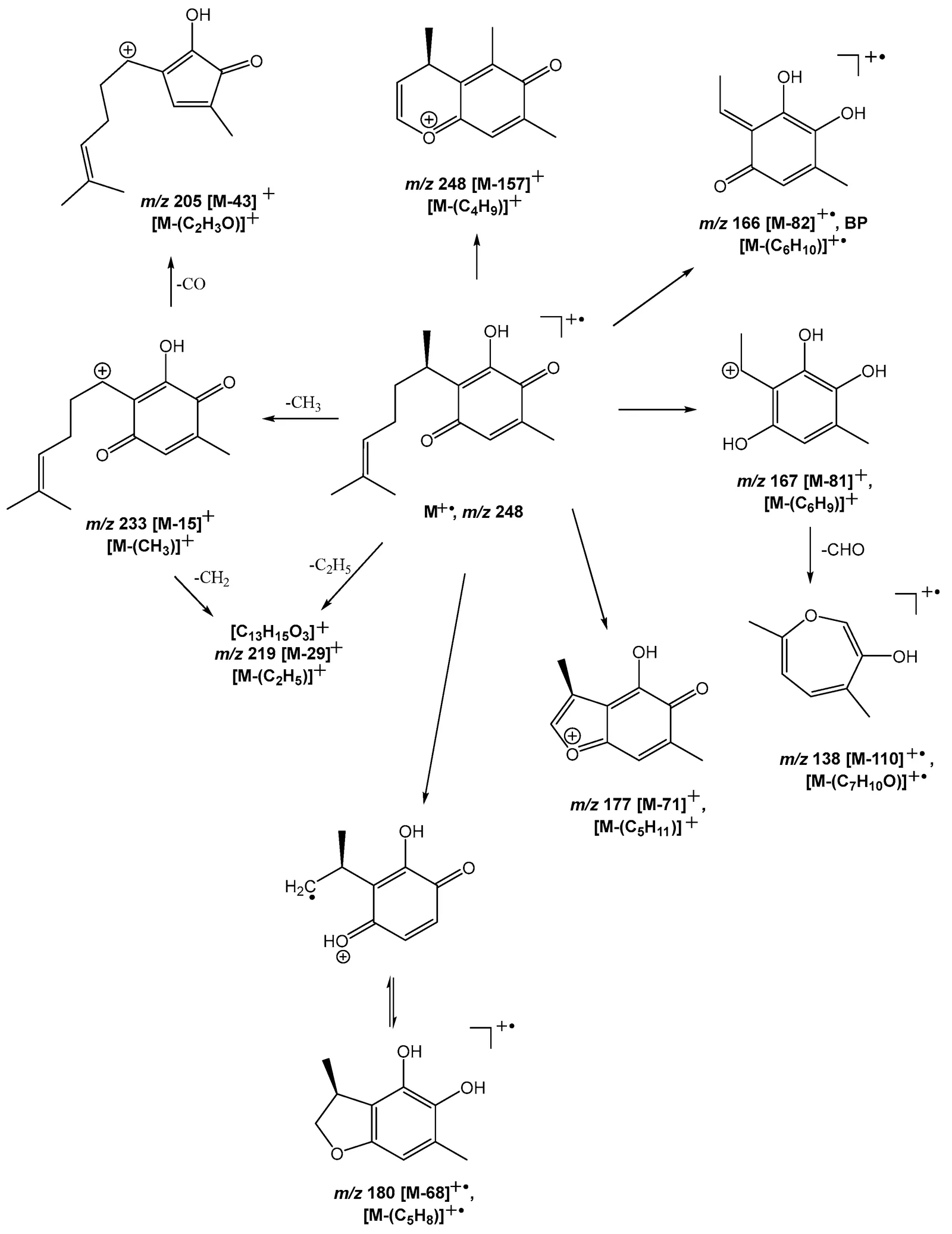
Electron ionization, fragmentation pattern of **1** and several of its analogs. Adapted from Ref. [21].

In Ref. [21], the authors reported an EI-MS study of perezone (**1**) in comparison with several of its derivatives, including isoperezone (**2**), α-pipitzol (**3**), β-pipitzol (**4**), and β-isopipitzol (**6**), establishing a common fragmentation pattern (Scheme 1), albeit with different relative abundances (Table 2). For **3** and **4**, the molecular ion proved to be the base peak, whereas in the case of **6**, it was *m*/*z* 167.

According to the established fragmentation pattern, respective fragment ions [M-15]^+^ are achieved from the corresponding molecular ions. Further fragment ions, [M-43]^+^, were formed from [M-15]^+^ by the subsequent loss of CO. Fragment ions [M-57]^+^, [M-68]^+•^, and [M-71]^+^ were formed from the molecular ion through the loss of C_4_H_9_, C_5_H_8_, and C_5_H_11_, respectively, from the side chain. It should be noted the fragment ions *m*/*z* 167 and 166 proved to be very abundant, originating from the removal of C_6_H_9_ and C_6_H_10_ from the side chain, respectively.

#### 3.1.12. Structure of α-Isopipitzol (4,8,8,10-Tetramethyl-9-hydroxy-2,12-dioxotricyclo[5.3.1.0^3,7^]undec-1-ene) (2000)

The authors in [6] reported that perezone (**1**) could be converted into α-isopipitzol (**5**), a new molecule, along with two other tricyclic sesquiterpenoids, using activated ignimbrite in the presence of 10% HCl. Following chromatographic separation of the reaction mixture, **5** was recovered as a white solid and was purified by recrystallization from *n*-hexane/ethyl acetate (8:2).

The mechanism of formation of α-isopipitzol (**5**) was explained as a dipole 1,3-addition, a pathway distinct from those leading to α- and β-pipitzols (**3** and **4**, respectively).

**Figure 10:**
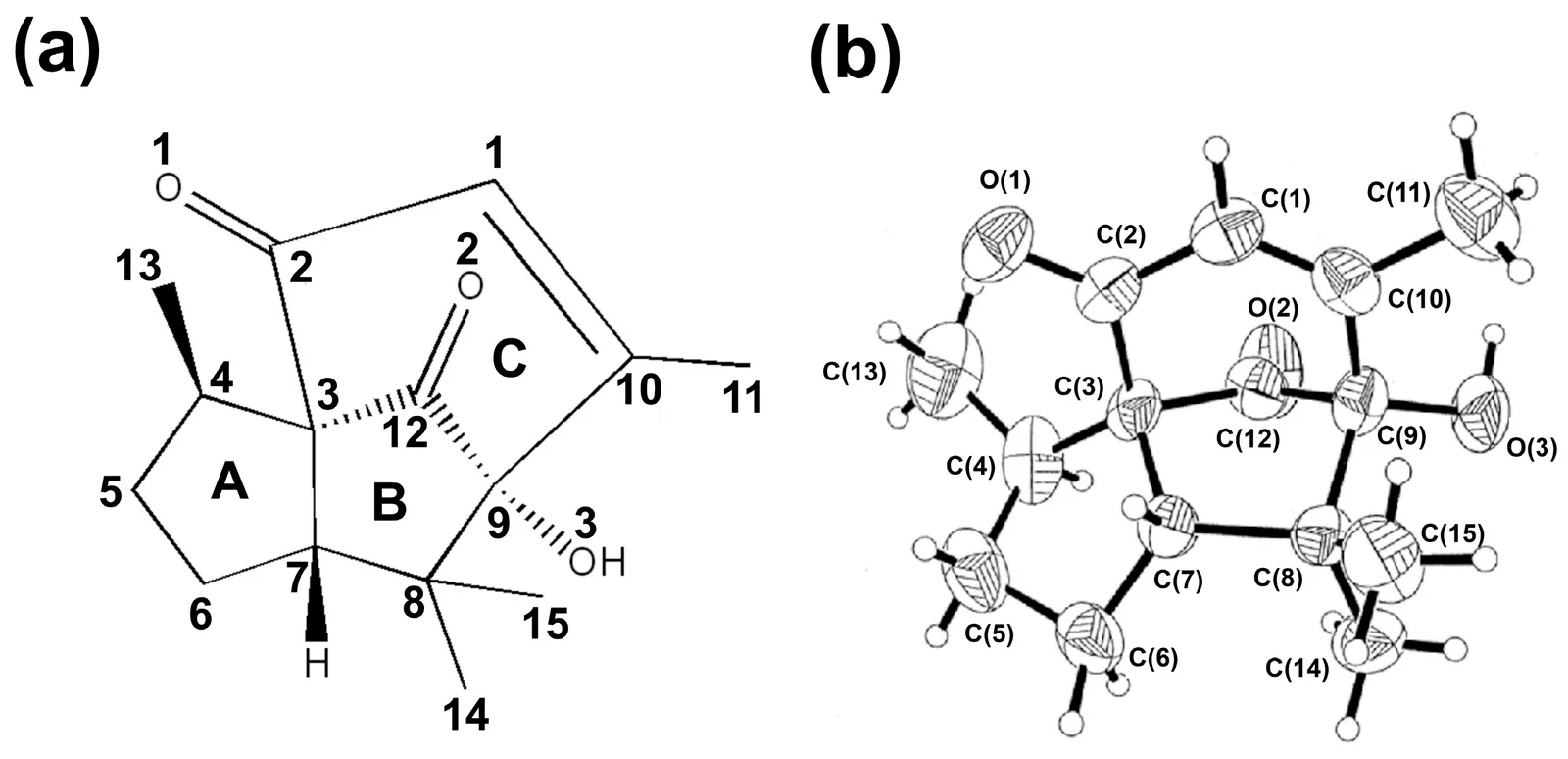
(**a**) Structure and (**b**) XRD of α-isopipitzol (**5**).

The structure of **5** (Figure 10a) was unequivocally established by single-crystal X-ray diffraction analysis (Figure 10b). The molecule has two five-membered *cis* rings (A and B) fused at C3-C7 in envelope and half-chair conformations, respectively, and a six membered C ring that is fused with the B ring at C3-C12-C9 and adopts a distorted envelope conformation. The hydroxyl group at C9 forms a hydrogen bond with O1 of another molecule, resulting in the formation of infinite chains along the b-axis that stabilize the crystal.

### 3.2. Synthesis and Stereoselectivity

#### 3.2.1. Structures of α- and β-Pipitzols (1965)

It has been established that pipitzol is a thermal product of perezone (**1**). Although it was apparently obtained as a homogeneous product, its spectroscopic data evidenced a mixture of two sesquiterpenic stereoisomers, α- and β-pipitzols (**3** and **4**, respectively) [3]. Comparing the ^1^H NMR spectrum of **1** with those of the pipitzols, it became clear that the side chain and quinone double bonds of the enedione moiety of **1** were involved in the formation of pipitzols. Both stereoisomers are weak acids, providing positive results in FeCl_3_ testing. Both pipitzols show a band in their infrared spectra at 1760 cm^−1^, attributable to a carbonyl group of a five-membered cyclic ketone, and bands at 3450, 1670, and 1635 cm^−1^, attributable to an enolized six-membered α-diketone. In OCD spectra of the enantiomeric pipitzols, **3** shows a strong positive Cotton effect, whereas **4** shows a negative Cotton effect. Their structures are related to the cedrene skeleton.

#### 3.2.2. Total Synthesis of Perezone and α- and β-Pipitzols (1965)

**Scheme 2:**
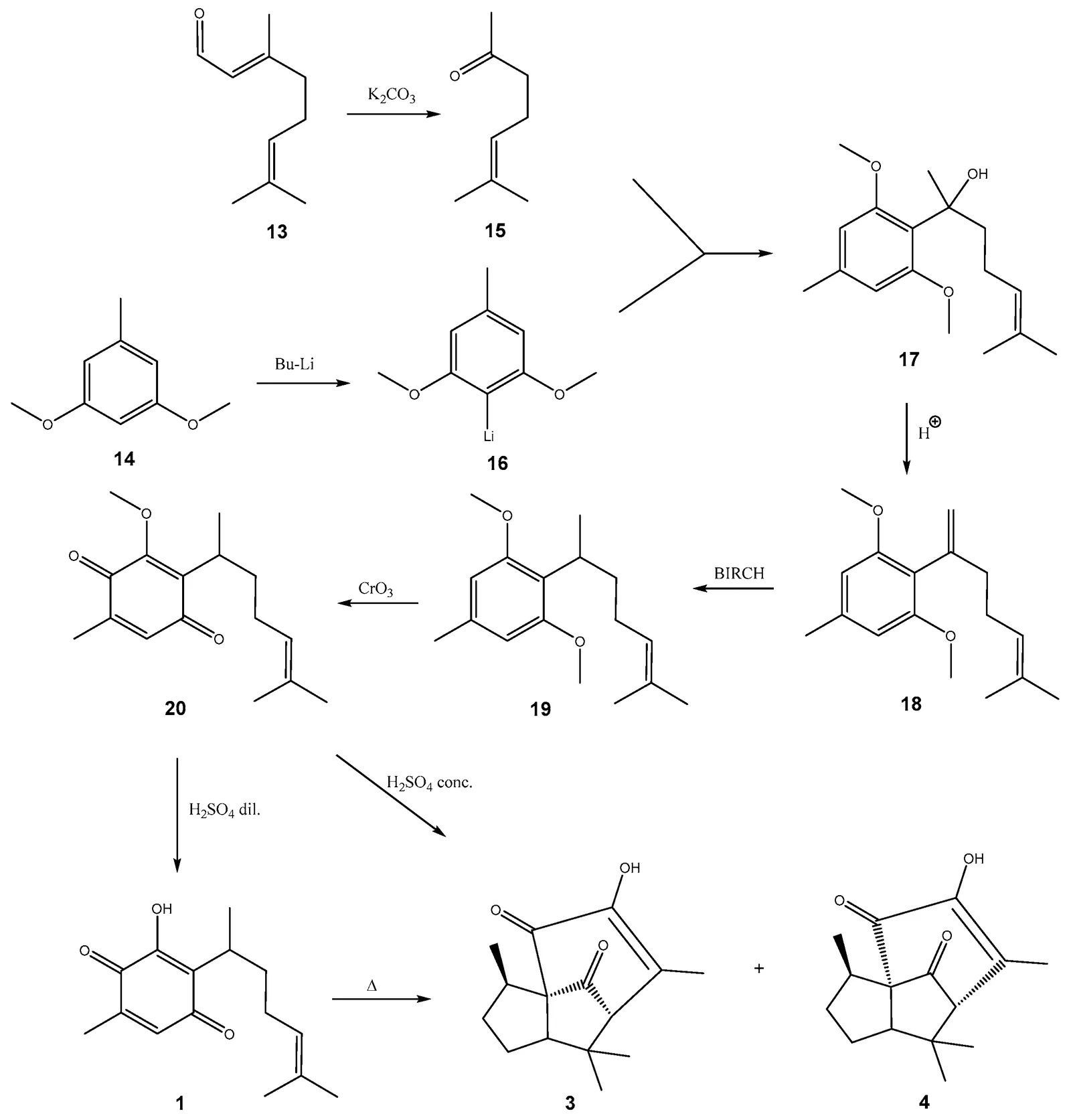
*Rele* synthesis of perezone and α- and β-pipitzols (**3** and **4**, respectively). Adapted from Ref. [22].

The first synthesis of **1**, **3**, and **4** is summarized in Scheme 2 [22]. The starting materials are geranial (**13**) and 3,5-dimethoxytoluene (**14**), and several intermediate products (**15**–**19**) are involved, implying a convergent synthesis. Since the production of methoxyperezone (**20**) proceeded with a very low yield (7.75%), we decided to continue the reaction by employing **20** obtained through the methylation of perezone instead of synthetic methoxyperezone (*rele*). The intermediate and final products were characterized spectroscopically. The 1H NMR spectrum of **3** in CDCl_3_ features a broad signal at 6.31 ppm, attributable to the hydroxyl group; two singlets at 2.78 and 2.06 ppm, attributable to the hydrogen adjacent to the carbonyl group and those of the methyl group adjacent to the double bond, respectively; a doublet at 1.34 ppm due to the methyl group attached to the cyclopentane ring; and a singlet at 1.09 ppm due to the two methyl groups attached to the other cyclopentane ring.

#### 3.2.3. Studies of Perezone Derivatives, Structures of Pipitzols and Perezinone (1966)

The medicinal properties of some Mexican plants have motivated research on the constituents of many roots, including those of the *Perezia* family (*Acourtia*), particularly *Perezia cuernavacana*, from which **1**, **3**, and **4** were isolated [23]. Although pipitzol initially appeared to be a single product, its ^1^H NMR spectrum showed the duplication of some signals, indicative of a mixture. After isolating pipitzol from the mother liquor, a benzoate was prepared that showed two distinct boiling points. After careful alkaline hydrolysis, an isomer of **3**, β-pipitzol (**4**), was identified. In OCD spectroscopy, **3** displayed a positive Cotton effect, while **4** displayed a negative effect.

#### 3.2.4. Further Studies on Hydroxyperezone Derivatives (1974)

**Figure 11:**
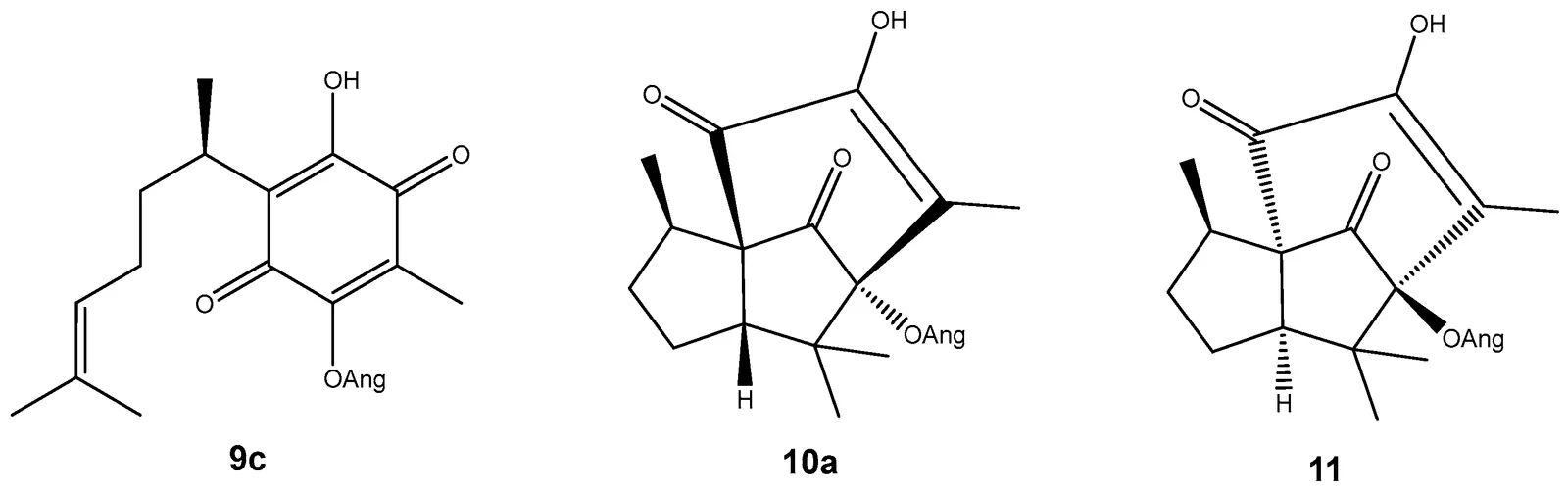
Structures of hydroxyperezone monoangelate (**9c**), α-perezol (**10a**), and β-perezol (**11**).

The position of the angeloyl group of *O*-angelohydroxyperezone (**9c**) was established following its thermolysis, which generated a mixture of α- and β-perezols (**10a** and **11**, respectively) (Figure 11) [24]. The spectroscopic properties of these perezols were proven to be essentially those of pipitzols plus those of the angeloyl group. In ORD curves, **3** and **18a** exhibited a positive Cotton effect, whereas **4** and **19** exhibited a negative Cotton effect.

#### 3.2.5. The Reaction Mechanism of Perezone–Pipitzol Transformation (1977)

**Scheme 3:**
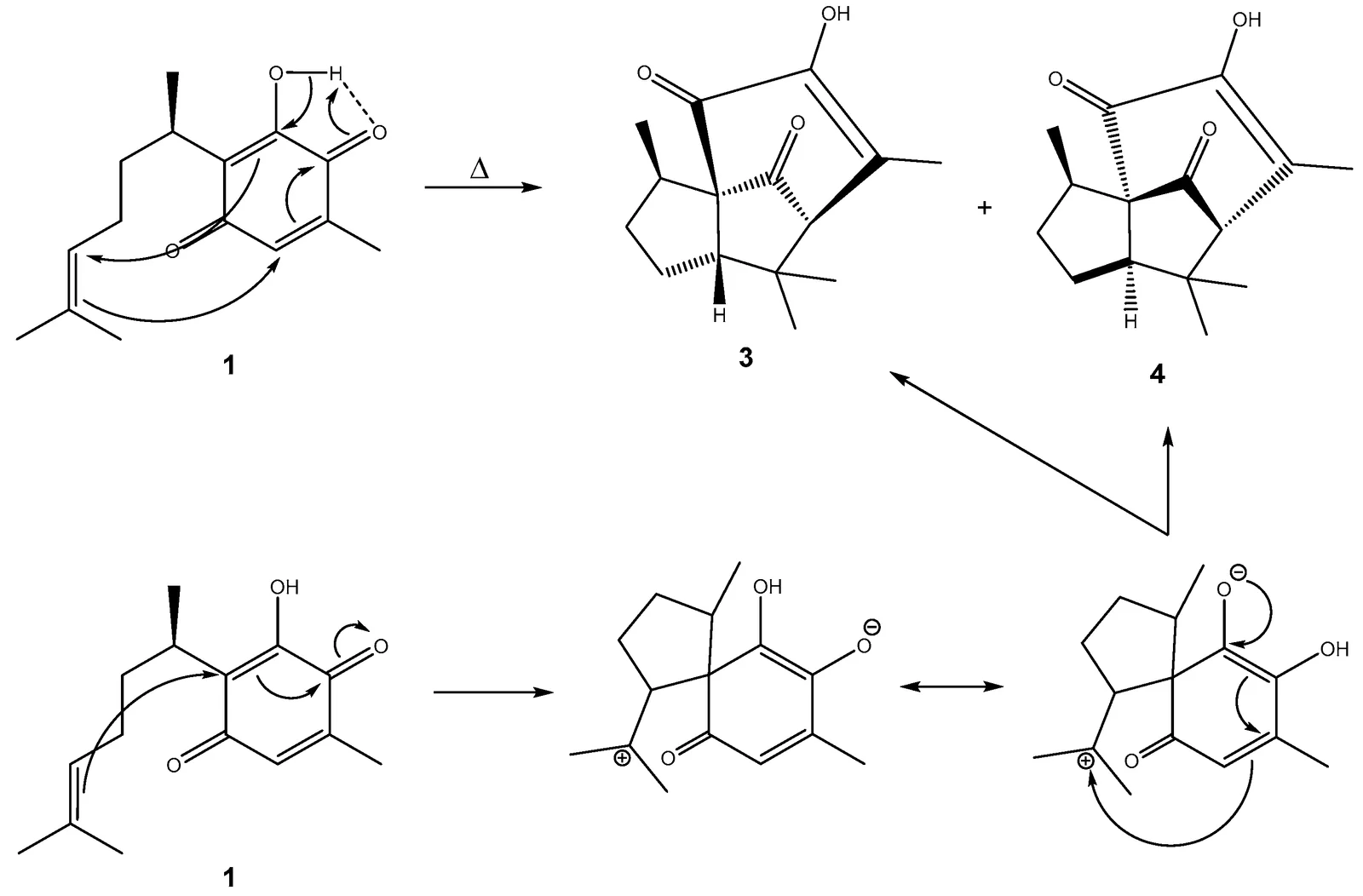
Proposed reaction mechanism to transform **1** into **3** and **4**. Adapted from Ref. [25].

After the isolation and characterization of α- and β-pipitzols, **3** and **4**, respectively, two possible reaction mechanisms were proposed for the corresponding thermal transformation of perezone (**1**) [25] (Scheme 3).

**Figure 12:**
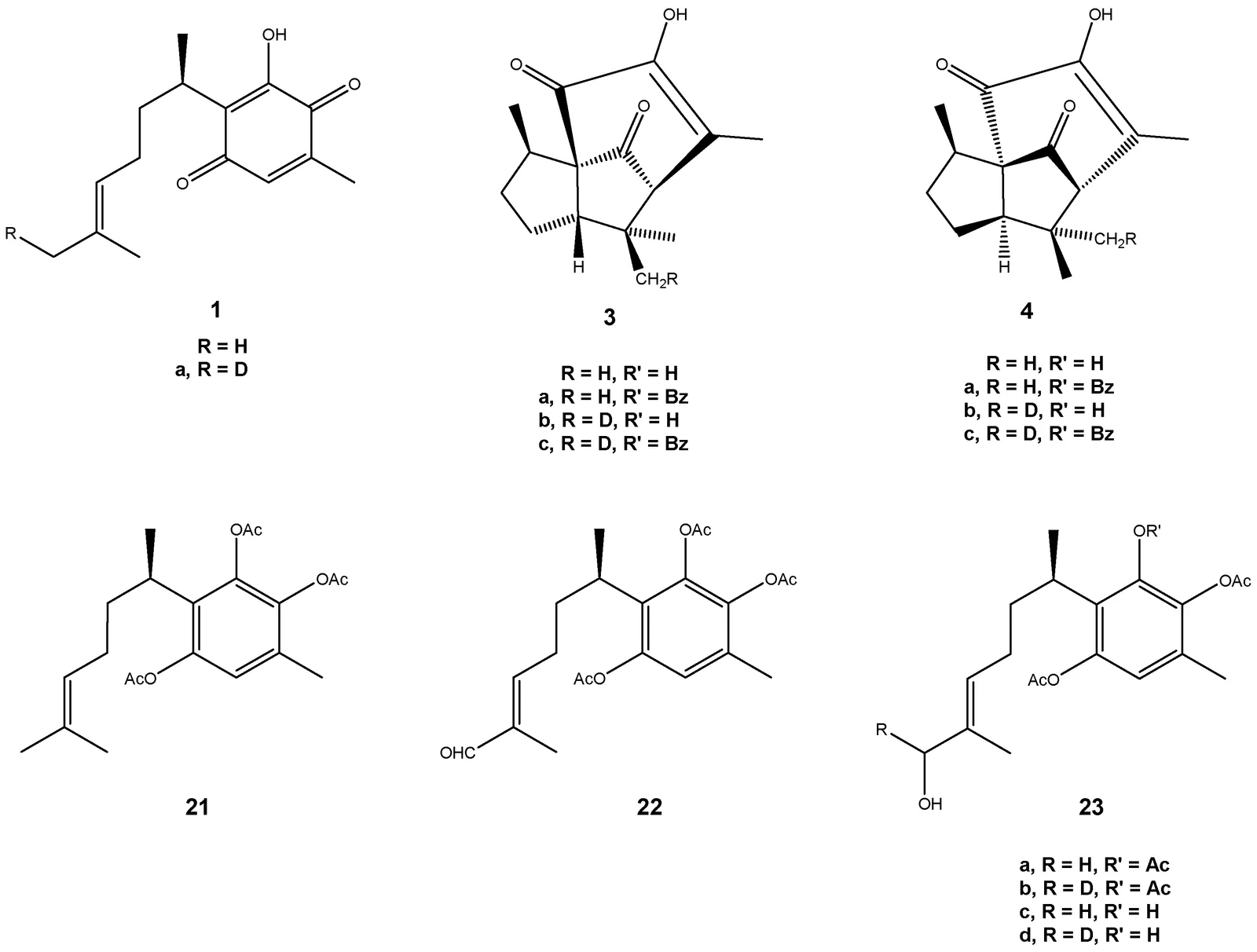
Products during the transformation of **1** into **3** and **4**. Adapted from Ref. [25].

From several transformations (Figure 12) performed on perezone (**1**), it was evidenced that one methyl group of the side chain could be functionalized and subsequently restored, thus permitting deuterium labeling as a means of establishing regioselectivity.

The treatment of **21** with sulfur dioxide produced **22** and **23a**, the reduction of which with sodium borodeuteride produced a mixture of **23b** and **23d**. These compounds were separated and treated with pyridine sulfur trioxide complex, producing monodeuterated perezone (**1a**).

#### 3.2.6. The Stereochemistry of Pipitzols and Perezols (1980)

The stereochemistry of **3** was established in [17] by transforming it into cedrene, and the study was complemented by the performance of a single-crystal X-ray diffraction analysis of α-pipitzol benzoate (**3a**). Furthermore, the stereochemistries of cedrenolides related to **4**, and α-, β-, and γ-perezols (**10a**, **11**, and **10b**, respectively) were appropriately assigned from their respective ORD curves.

**Figure 13:**
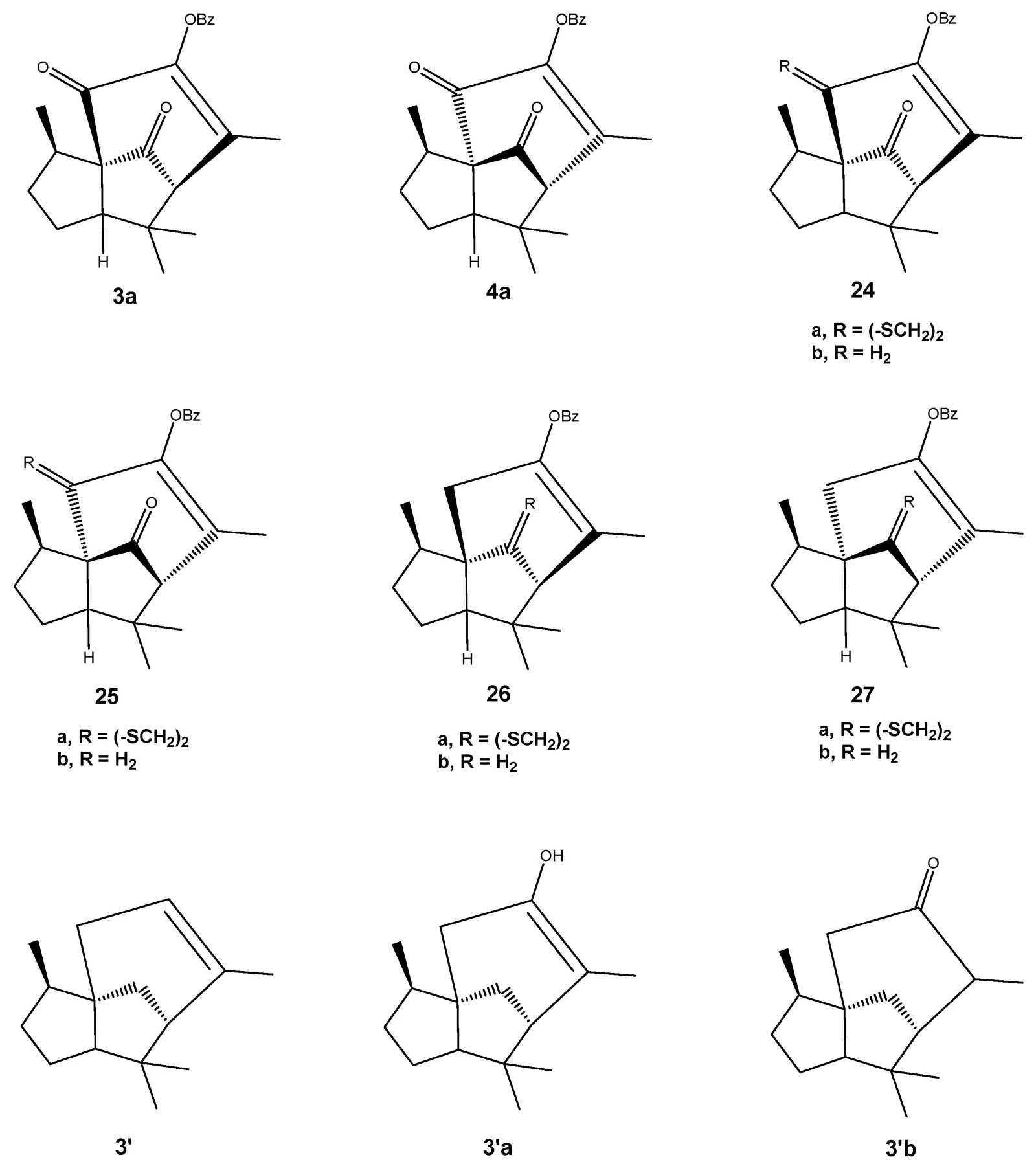
Structure of compounds involved in the study. Adapted from Ref. [17].

In brief, the benzoates **3a** and **4a** were submitted to a synthetic pathway generating various intermediate products: first the dithioketals **24a** and **25a**, followed by 4-desoxo-α-pipitzol-benzoate (**24b**) and 4-desoxo-β-pipitzol-benzoate (**25b**), and then the dithio derivatives **26a** and **27a** and the enol benzoates **26b** and **27b** (Figure 13).

Moreover, to establish which compound was derived from cedrene (**3’**), the double bond of the hydrocarbon was epoxidized, and a rearrangement with boron trifluoride produced cedrenone (**3’b**), which was treated with benzoic anhydride and perchloric acid to produce enol benzoate **26b**. Both samples were identified as **27b** by UV and IR spectrophotometric data; however, significant differences were observed in their ^13^C NMR spectra.

**Figure 14:**
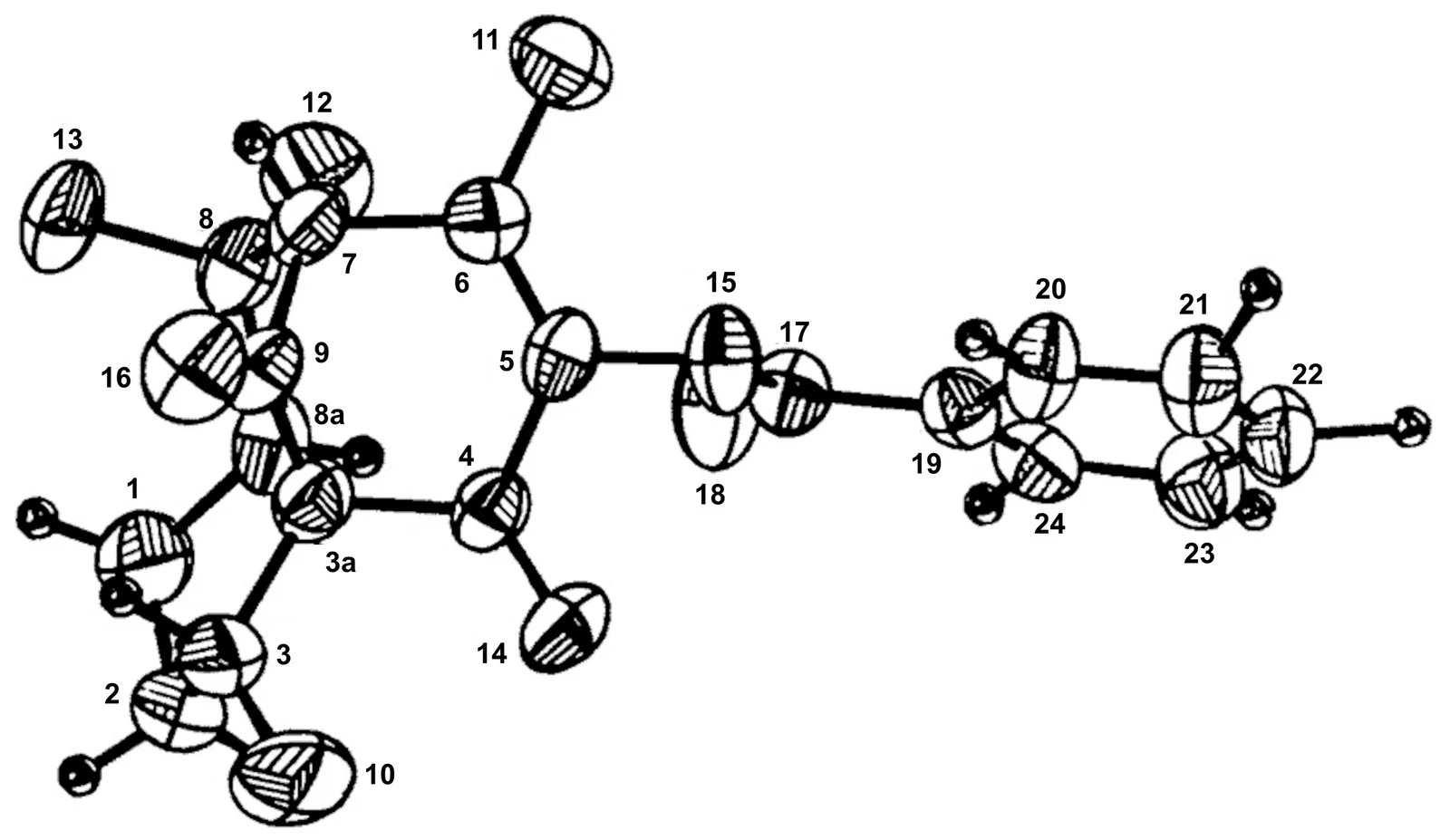
Single-crystal X-ray diffraction of α-pipitzolbenzoate (**3a**). Adapted from Ref. [17].

It is also important to highlight that the X-ray crystallographic diffraction data for α-pipitzol benzoate (**3a**) revealed the conformation of its complete structure (Figure 14). Both α- and γ-perezols (**10a** and **10b**, respectively) showed a positive Cotton effect, with stereochemistry akin to that of **3**, while β-perezol (**11**) showed a negative Cotton effect, with stereochemistry akin to that of **4**.

#### 3.2.7. Reaction Mechanism Change in the Lewis-Acid-Catalyzed Perezone–Pipitzol Transformation (1981)

The formation of pipitzols (α, β) from **1** has been explained as a [4+2] cycloaddition [26], highlighting the lack of stereochemical induction; both pipitzols are obtained in equimolar quantities. Nevertheless, it is possible to induce high stereoselectivity by suitably controlling the process conditions. Using eight equivalents of BF_3_ as a Lewis acid, at 0 °C, a mixture of **3** (90%) and **4** (10%) was produced; using 0.1 equivalents of BF_3_, 29% of both pipitzols and 30% of diperezone were formed.

#### 3.2.8. The Stereocontrol of the Perezone to Pipitzol Transformation (1984)

Having established that it is feasible to achieve the highly selective production of **3** from **1** using BF_3_, Ref. [27] reported that stereochemical control via moderating the temperature results in a major proportion of **4**.

Compound **4** was also the dominant product, in a 3:1 ratio, when applying AlCl_3_ and diethyl sulfide to *O*-methylperezone. The change in stereochemistry was rationalized by considering the difference in the transition state compared to that for **3**, e.g., a strong steric interaction between the secondary methyl group and the methoxyl groups.

#### 3.2.9. Formal Total Synthesis of β-Pipitzol (1985)

**Figure 15:**
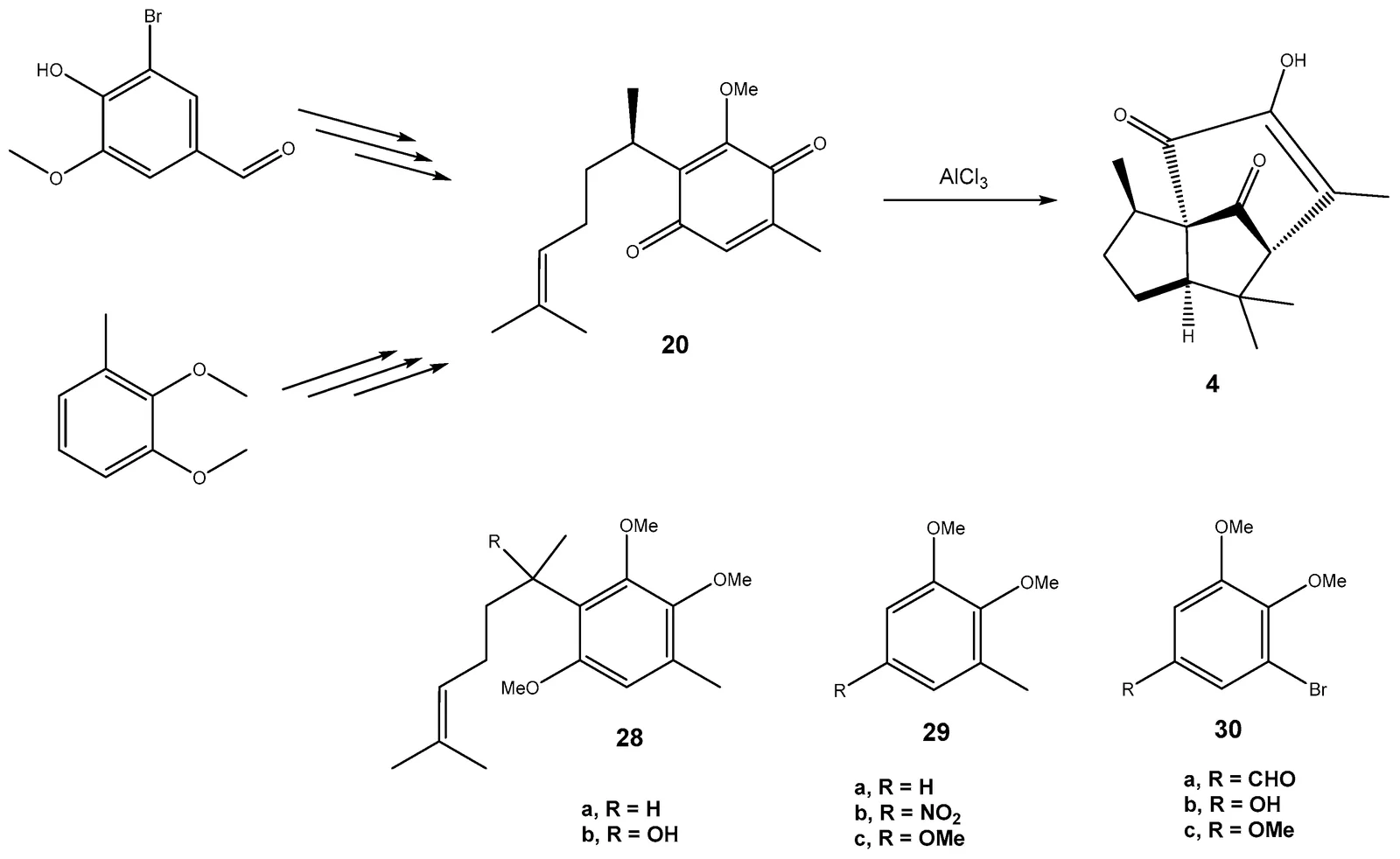
Formal-total synthesis of β-pipitzol (**4**) from the synthesis of *O*-methylperezone (**20**).

Sanchez et al. [28] first examined the stereocontrolled transformation of various substrates to mixtures of α- or β-pipitzols (**3** and **4**, respectively) and its dependence on the substrate and the reaction conditions. Thus, the treatment of perezone (**1**) with boron trifluoride yielded α-pipitzol (**3**) as the main product; however, when using *O*-methylperezone (**14**) in the presence of aluminum trichloride, the β-isomer (**4**) was favored. Accordingly, the authors explored the use of *O*-methylperezone (**20**) to perform a formal total synthesis of β-pipitzol (**4**). They described the synthesis of **20**, which involved a silver (II) *O*-demethylation of the phenolic ether **20**, obtained by the condensation of 2,3,5-trimethoxytoluene with 6-methyl-5-hepten-one, followed by the removal of the tertiary alcohol group. The aromatic precursor was synthesized through two alternative routes starting from either commercially available 5-bromovanillin or 3-methylveratrole. The reaction intermediates along these routes are depicted as **28**–**30** in Figure 15.

#### 3.2.10. Contribution to the Chemistry of β-Pipitzol (1985)

**Scheme 4:**
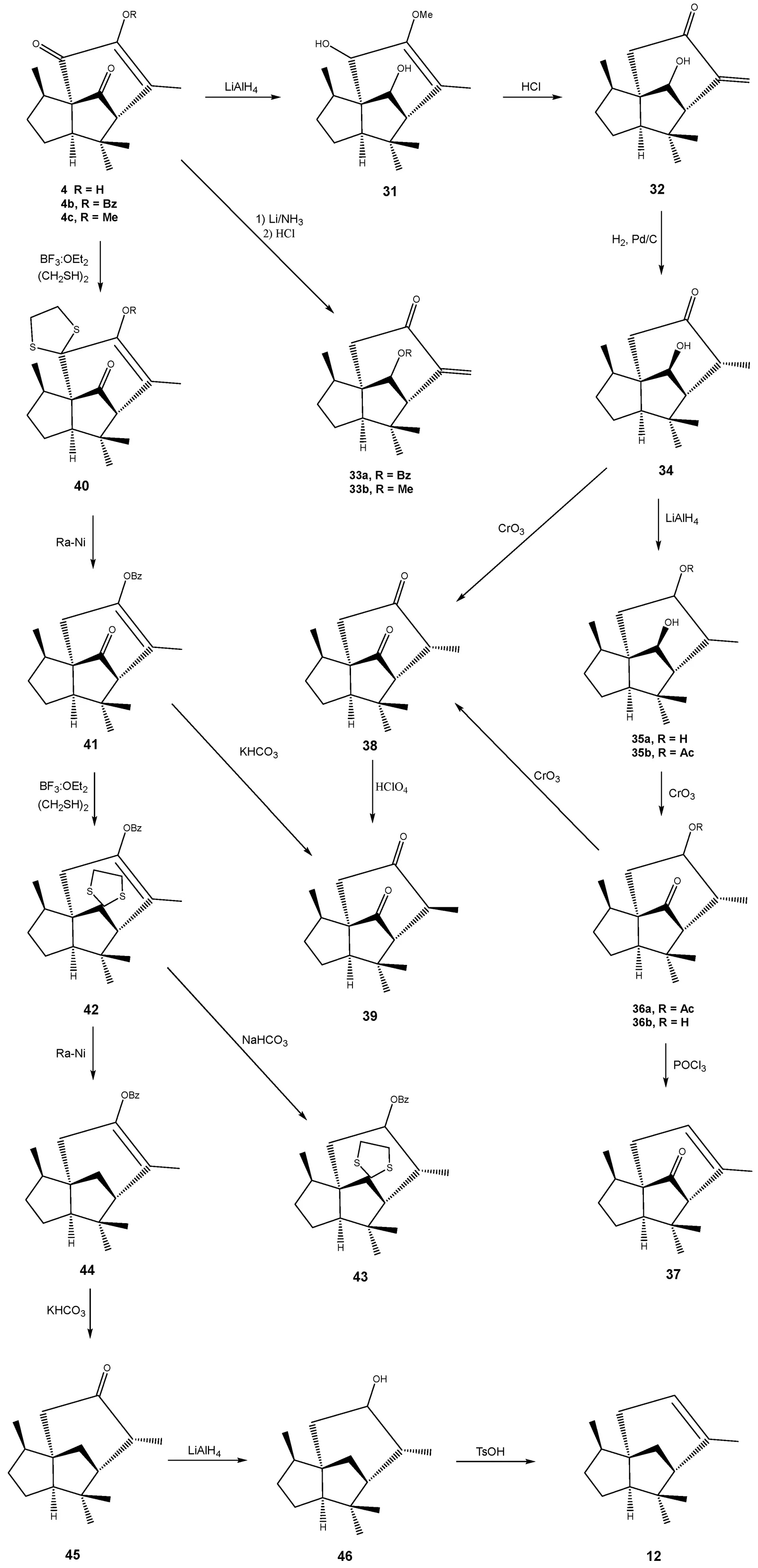
Derivatives **4b**–**c** and **30**–**46** of β-pipitzol (**4**). Adapted from Ref. [30].

At the time of this research, the chemistry of **4** was not well characterized and so various chemical transformations were explored (Scheme 4) [29]. The study led to novel derivatives of **4**, correlating the target molecule with ent-3-epi-α-cedrene (**12**), commonly known as 3-epi-α-cedrene. Alcohol **31**, obtained from *O*-methyl-β-pipitzol (**4c**) via reduction with LiAlH_4_, was subjected to 1,4 dehydration by acid treatment, generating ketoalcohol **32**; this differs in stereochemistry at C9 with respect to ketoalcohol **33a** obtained from **4c** with Li/NH_3_. The hydrolysis of benzoates **41** and **44** produced ketones **39** and **45**, respectively, differing in stereochemistry at C6. The catalytic hydrogenation of ketoalcohol **32**, followed by oxidation, generated diketone **38**, an epimer of **39** at C6, **as** deduced by the corresponding ORD curves. Spectroscopic data and unambiguous stereochemical assignments were provided for all compounds.

#### 3.2.11. BF_3_-Catalyzed Cycloadditions of Naturally Occurring Sesquiterpene p-Benzoquinones (1986)

Cycloadditions have become a subject of increasing interest due to their synthetic versatility. However, intramolecular cycloadditions, such as those involved in perezone–pipitzol transformation, have not been extensively studied. This thermal reaction seems to combine the characteristics of a sigmatropic order change [1,9] and an ionic B-type [4+2] cycloaddition.

The information obtained indicated that the substituents on the rings determine the outcome and even the stereoselectivity.

The results of the work in [30] revealed that the stereochemistry and regiochemistry of such cycloadditions are very sensitive to electronic and steric factors. Although no attempt was made to delineate the effect of varying the Lewis acid, the effect of substitution at C15 in **1** and **17a** was analyzed. It was established that hydroxyquinones exhibit complex and unpredictable behavior in an acidic environment, and that BF_3_ is a viable catalyst for the formation of pipitzol analogs in cases where the 6-position of the quinone is free or protected by a hydroxyl group.

It was expected that the cyclization of *O*-angeloylperezone would predominantly form **4**, and indeed **4** and *O*-angeloyl-β-pipitzol were obtained in a 9:1 ratio.

Analysis using the Dreiding model suggested that a 3-*O*-substituent larger than hydrogen would interact with the secondary methyl group, favoring the formation of **3**; since this interaction is relieved if the approach occurs on the opposite side of the ring, **4** is favored.

As a result, even small changes in the structure of quinones can lead to a huge change in the reaction outcome. The processes catalyzed by BF_3_•Et_2_O involve initial coordination of the catalyst, which, in turn, induces polarization of the conjugated enone system by promoting 1,4-additions whenever the 6-position is free or protected by an alcohol. On the other hand, substitution at the 15-position causes a perturbation of the molecular orbital of ethylene, probably by increasing the HOMO-LUMO gap energy and favoring an alternative pathway. Finally, based on the stereochemistry of the products of perezone and *O*-angeloylperezone, it is inferred that steric factors play a key role.

#### 3.2.12. A New Approach to the Cedrane Ring System via Intramolecular [4+2] Tropone-Olefin Cycloaddition: Total Synthesis of (±)-α- and β-Pipitzol (1987)

Generally, [4+2] cycloadditions produce *endo* cycloadducts with a skeleton related to bicyclo[5.3.0]decan or bicyclo[3.2.2]nonane; these cycloadducts may feasibly be transformed into ring systems present in natural products. For example, the cleavage of one carbon atom from the two-carbon olefin bridge could form bicyclo[3.2.1]octane, which is a subunit of cedrane-like compounds [31].

**Scheme 5:**
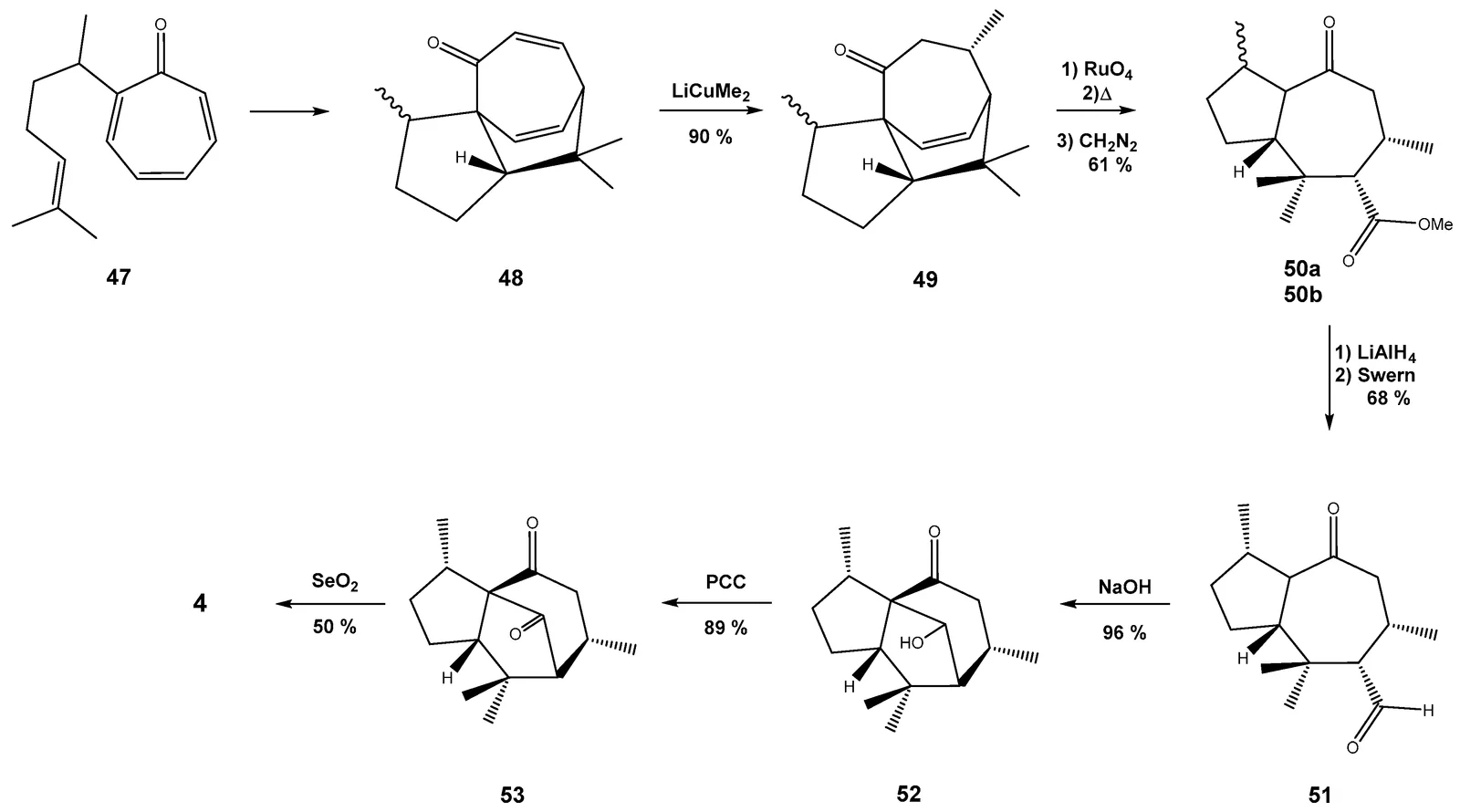
Synthetic route of the pipitzols. Adapted from Ref. [31].

The diastereoisomeric mixture of enone **48** generated from the [4+2] cycloaddition of tropone **47** proved to be viable for the formation of pipitzols. First, it was necessary to transform the bicyclo[3.2.2]nonane into a bicyclo[3.2.1]octane. To this end, the conjugate addition of lithium dimethylcuprate to **48** formed ketone **49** with a high degree of stereoselectivity. Next, oxidative cleavage of the double bond of **49** and subsequent decarboxylation were performed to remove the bridge. Esterification of the keto acids resulted in ketoesters **50**. Subsequently, the reduction and re-oxidation of **50a** provided ketoaldehyde **51**, and then the bridge of the bicyclic system was reconstructed with aqueous NaOH under reflux, resulting in aldol **52**. The oxidation of **52** yielded crystalline dione **53**, and subsequent oxidation with SeO_2_ furnished (±)-β-pipitzol, which was spectroscopically identical to an authentic sample. The diastereoisomer **50b** was converted to (±)-α-pipitzol under the same reaction conditions as applied for **50a**. The important points emerging from this work are summarized in Scheme 5.

#### 3.2.13. Carbohydrates to Carbocycles: A Synthesis of (−)-α-Pipitzol (1990)

**Figure 16:**
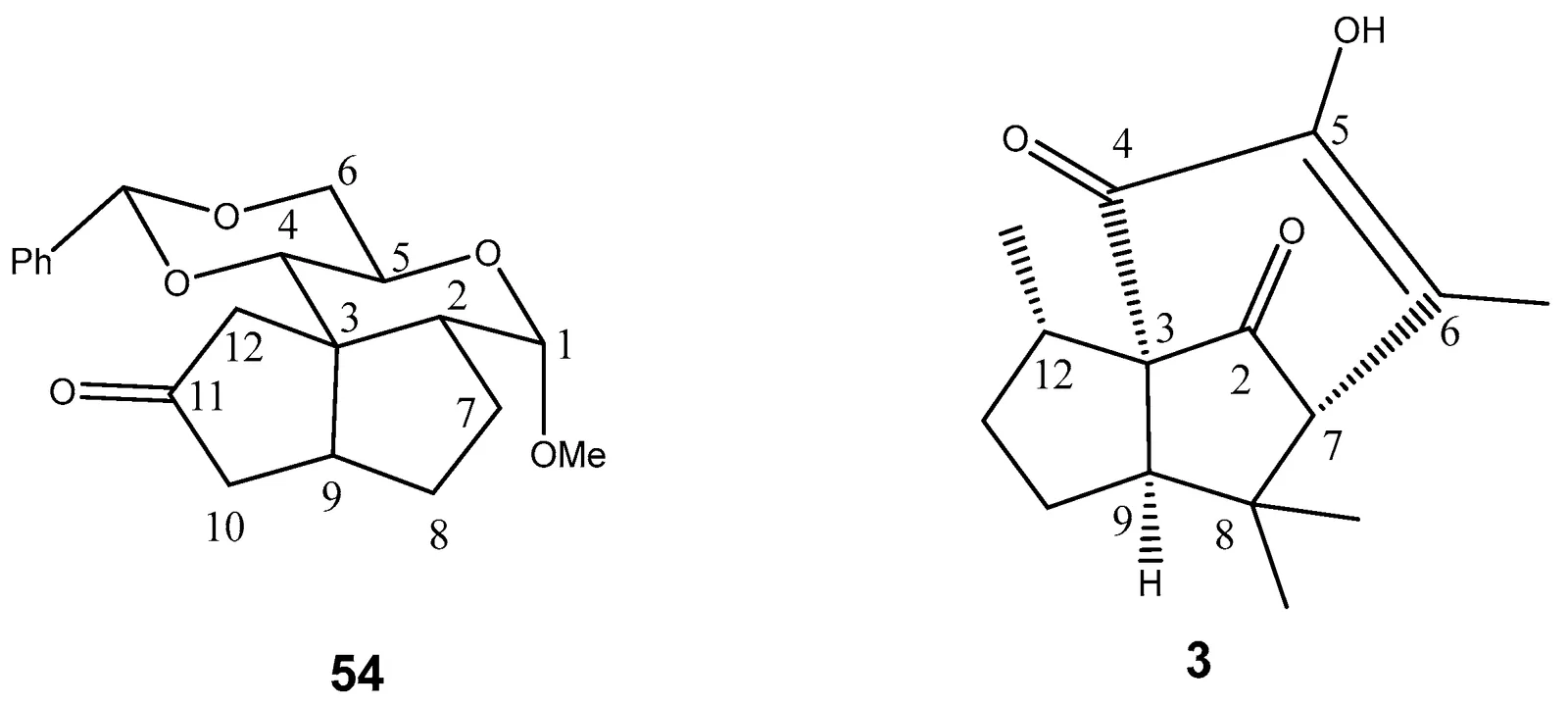
Structural correlation between pyranosidodiquinane (**54**) and **3**. Adapted from Ref. [32].

In this work [32], a strategic synthetic plan was proposed to obtain α-pipitzol (**3**) from a sugar derivative. The authors reported that a 2-deoxi-3-keto sugar could undergo serial radical cyclization to produce the key intermediate pyranosidodiquinane (**54**; Figure 16). They noted that the connectivity between carbon atoms C-6 and C-7 and the methyl groups could be introduced in further stages of the synthesis.

**Scheme 6:**
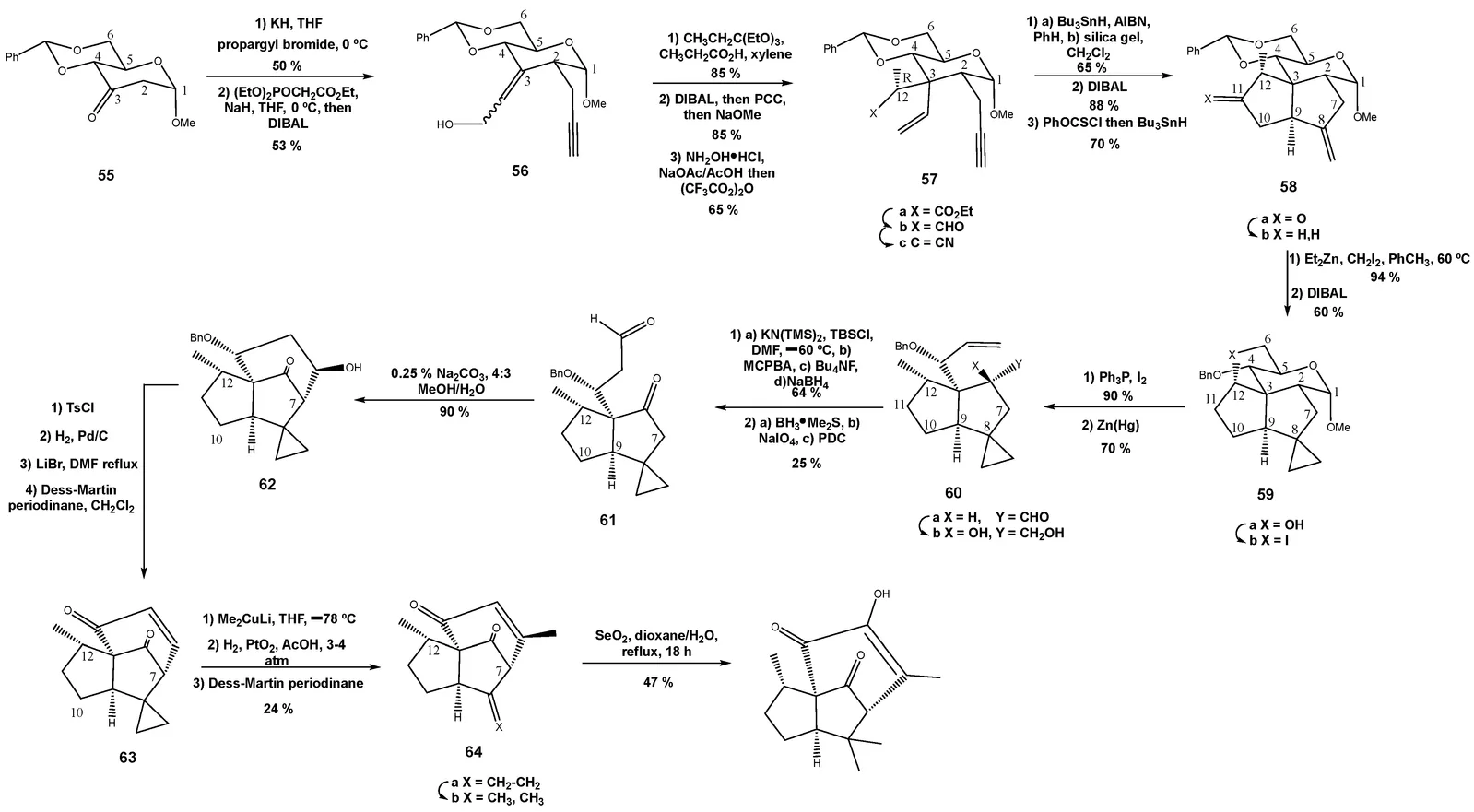
Synthesis of (−)-α-pipitzol from sugar. Adapted from Ref. [32].

In a complementary study, C-12 was added by a spiro Claisen rearrangement of **56** (Scheme 6). C-8 was implemented by *gem*-dimethyl substitution from radical cyclization of the nitrile derivative **57c**, whereby **58a** was produced as a single isomer. A central feature was a synthon on the C-2 carbonyl group, vicinal diol **60b**, generated by the Rubottom reaction. Further, the cleavage of the cyclopropane ring followed by re-oxidation of the secondary alcohol yielded **64b** in a chiral nonracemic form. Finally, oxidation with SeO_2_ yielded (−)-α-pipitzol.

#### 3.2.14. Further BF_3_•Et_2_O-Catalyzed Cycloadditions of Sesquiterpenic p-Benzoquinones (1993)

When considering the research in [33], it is necessary to keep in mind three important aspects: (a) the thermal transformation of perezone (**1**) to **3** and **4** had been known for more than a century (at the time when the work was carried out); (b) there is a deficiency of stereochemical induction by the chiral center of the perezone; and (c) subsequent studies have shown that it is possible to direct the course of the reaction toward one of the two isomers depending on the conditions and the starting substrate; the influence of the substituents is also considerable due to the large number of products potentially generated in the cycloadditions.

**Figure 17:**
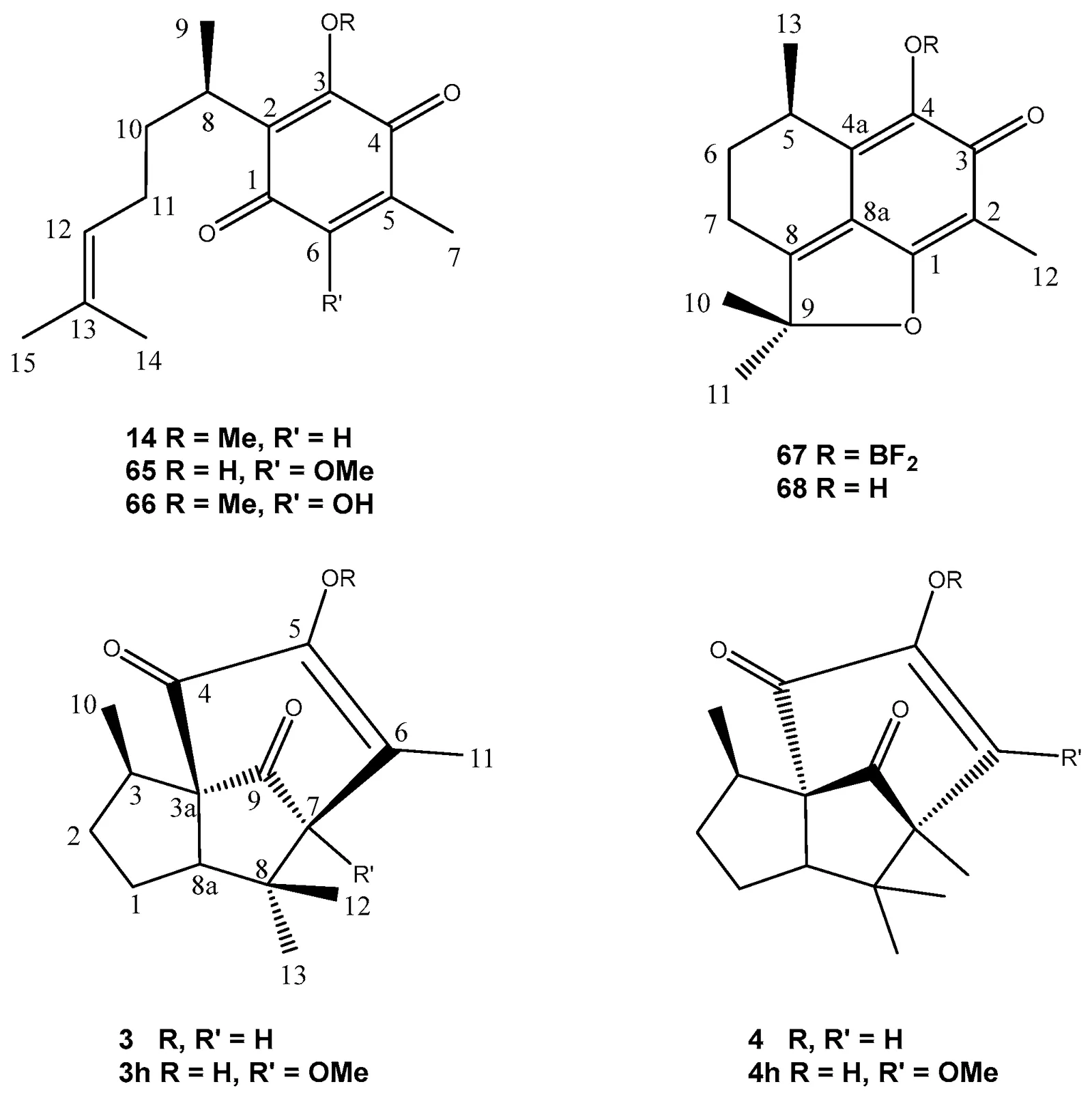
Structures of pipitzols and their derivatives. Adapted from Ref. [33].

As summarized in Figure 17, the results of this work allow the following statements to be made. Reactions of **20** in anhydrous CH_2_Cl_2_ at 0 °C and −20 °C in the presence of BF_3_•Et_2_O generated **3** and **4**, with **4** being the major product. The reaction of *O*-methyl-6-methoxyperezone with BF_3_•Et_2_O produced a mixture of 7-methoxy-α-pipitzol (**3h**) and 7-methoxy-β-pipitzol (**4h**), with the β-isomer again prevailing in both cases. In addition, a positive result was obtained from the FeCl_3_ test, demonstrating the presence of a methoxyl group at position 7.

The stereochemistries of **3h** and **4h** were determined by comparing the ORD curves in the region 589–365 nm, previously described for **3** and **4**. In their ^1^H NMR spectra, **3h** showed a chemical shift difference of 0.06 ppm, while **4h** showed a chemical shift difference of 0.02 ppm, matching the differences shown by **3** and **4**. The stereochemistries of the products were further confirmed by X-ray crystallography.

It was suggested that the reactions catalyzed by BF_3_•Et_2_O produced pipitzols or derivatives thereof if the 6-position was free or protected by an alcohol. Substitution with a free hydroxyl group generated BF_2_•perezinone (**67**) and perezinone (**68**), although it could not be confirmed at the time. Moreover, 6-methoxyperezone (**65**) and *O*-methyl-6-hydroxyperezone (**66**) were separately reacted with eight equivalents of BF_3_•Et_2_O at 0 °C and −20 °C. While cyclization of **65** resulted in **3h** and **4h** in a ratio of 2.5 to 1.0 in favor of the α-isomer, **66** generated **68** and **67** in ratios of 1.7 to 1.0 and 2.0 to 1.0 in favor of **68**, validating the previous assumption.

Finally, the highest stereoselectivities and yields were obtained at −20 °C with a 1:16 ratio of quinone to BF_3_•Et_2_O, highlighting the influence of both factors.

#### 3.2.15. Detailed Studies of Perezone Rearrangements (1997)

Among the many contributions to the chemistry of perezone, it has been discovered that the reaction conditions allow the manipulation of both the stereoselectivity and the regioselectivity of the products, offering a means of creating new polycycles [34].

Of particular interest is the transformation of **1** into **2** in a 58% yield by using 3,4,5,6-tetrahydro-2-pyrimidinethiol in absolute methanol. By reacting **2** in the presence of a Lewis acid, **6** or dihydroisoperezinone—or a mixture of both—is obtained (by intramolecular cycloadditions), depending on the Lewis acid used.

#### 3.2.16. An Oxidative Dearomatization-Induced [5+2] Cascade Enabling the Syntheses of α-Cedrene, α-Pipitzol, and Sec-Cedrenol (2010)

Cedranoids comprise a group of naturally occurring sesquiterpenes [35], which includes α-pipitzol (**3**). Surveying the long history of its structural determination, the authors of this publication drew attention to its generation from perezone (**1**) [25] by means of [4+2] cyclization, and its enantioselective synthesis by more than 30 steps from α-D-mannopyranoside [32].

**Scheme 7:**
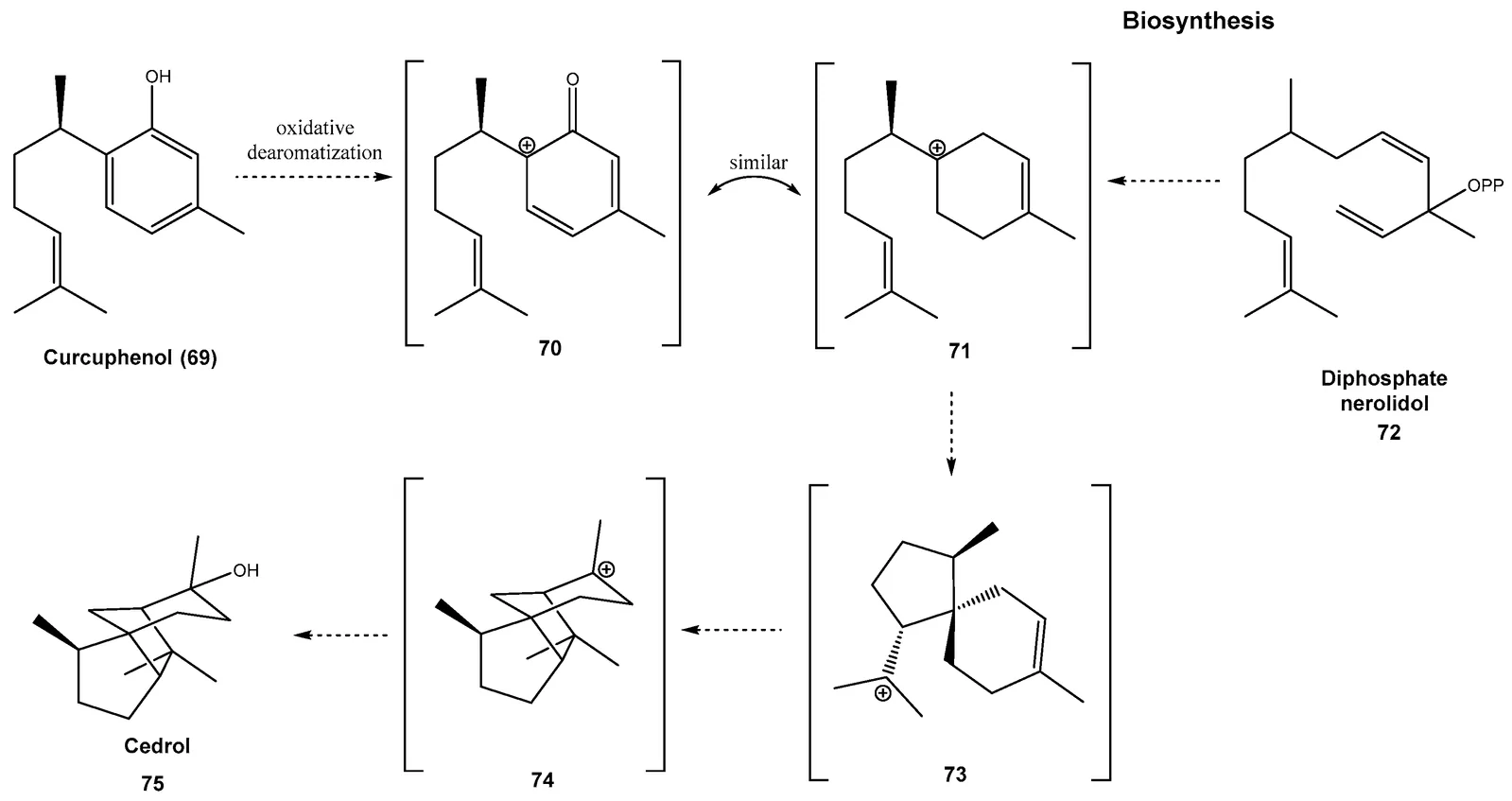
Biosynthetically inspired [5+2] cycloaddition. Adapted from Ref. [35].

Notably, for the preparation of the target molecules, the authors were inspired by the biosynthesis of the tricyclic skeleton of cedrol [36], as presented in Scheme 7.

**Scheme 8:**
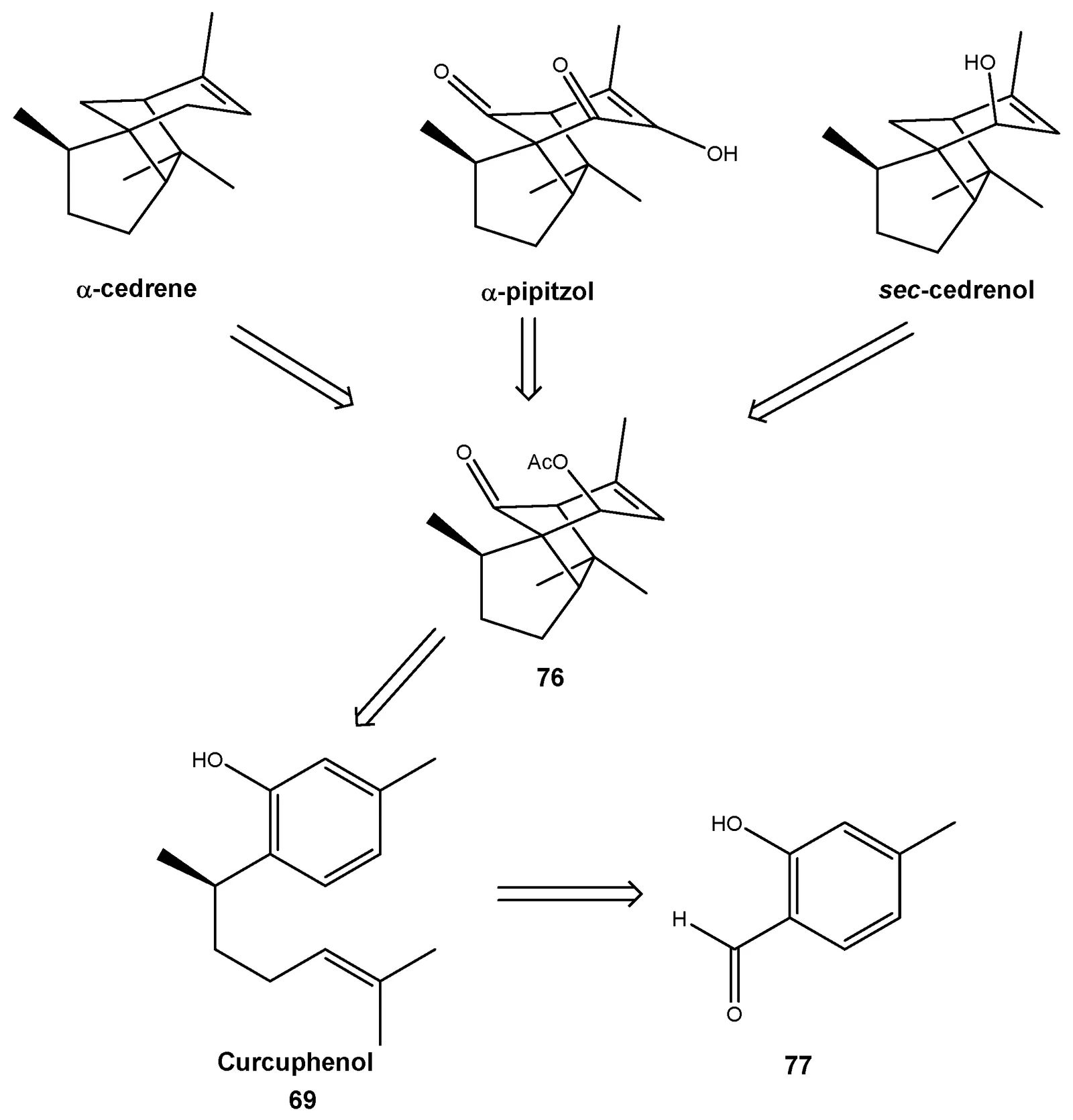
Retrosynthesis of the cedranoid family. Adapted from Ref. [35].

Once this research team was able to obtain curcuphenol (**69**), they used this approach to produce not only **3**, but also α-cedrene and *sec*-cedrene. The retrosynthetic analysis shown in Scheme 8 was designed for the target molecules.

It should be noted that this work was a modification of previous work carried out by the same research team, but using a salicylaldehyde derivative (**77**) [37,38,39,40]. Starting from curcuphenol **76**, a reaction cascade led to cedranoid-type compounds like **3**.

#### 3.2.17. A New Cedranolide from the FeCl_3_-Catalyzed Cycloaddition Reaction of Perezone

**Figure 18:**
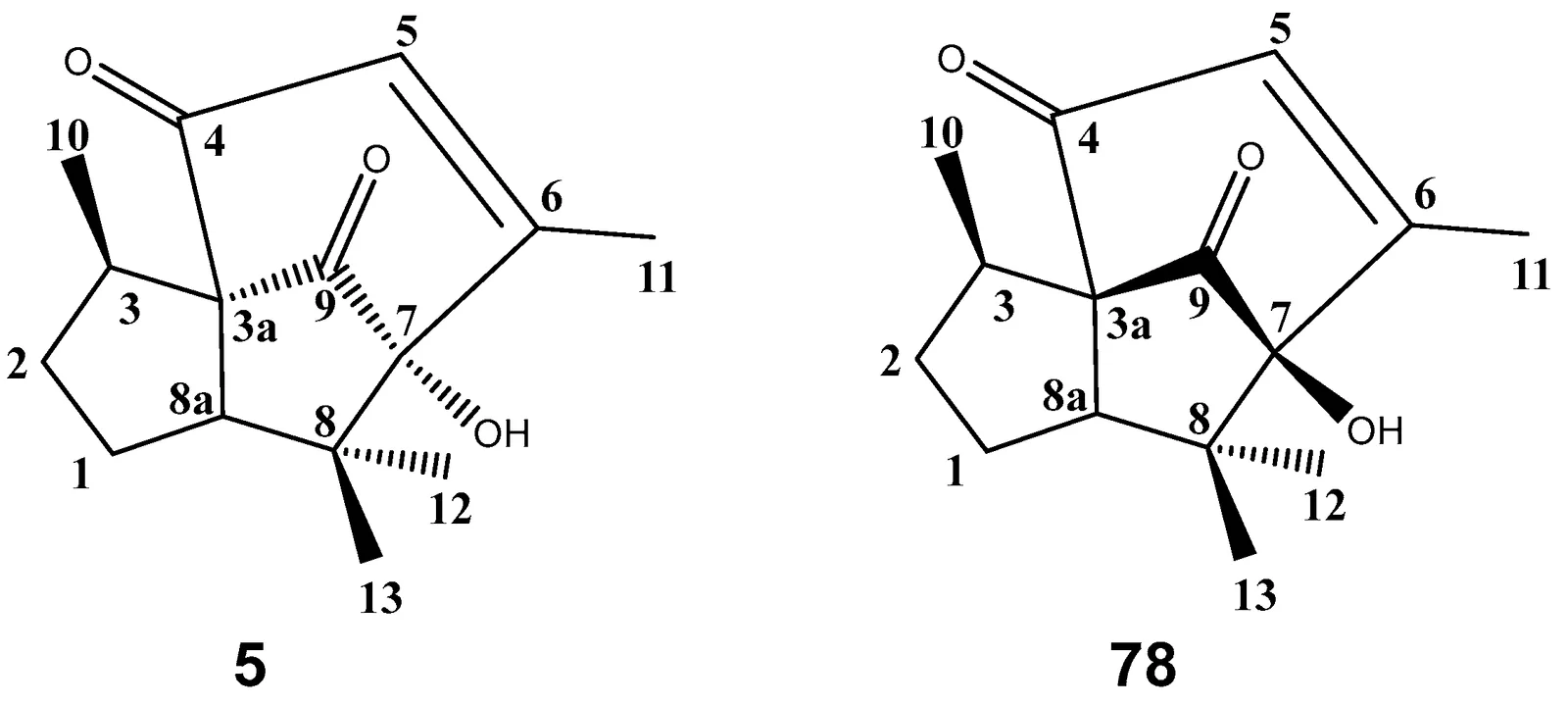
Structure of α-isopipitzol (**5**) and its inverse epimer (**78**).

The most recent publication related to the target compounds in this review [41] involves the FeCl_3_-catalyzed conversion of perezone, obtaining α-pipitzol (**3**), β-pipitzol (**4**), α-isopipitzol (**5**), and a novel molecule, (3a,7,8a)-tri-*epi*-α-isopipitzol (**78**) (Figure 18), considered the inverse epimer of **5**. The products were obtained in a 38:41:14:7 ratio. The absolute configuration of **5** was confirmed by VCD spectra, and the stereochemistry of its inverse epimer by the VCD chirality exciton approach, as (3*R*,3a*S*,7*R*,8a*S*) and (3*R*,3a*R*,7*S*,8a*R*), respectively.

The corresponding conformational explorations for **5** and **78** produced only two stable conformers for each epimer, in agreement with the elevated conformational strain characteristic of their structural frameworks. The relative orientation of the carbonyl groups in these conformers gives rise to characteristic bisignate VCD curves, whose Cotton effects agree with the dihedral angles observed in the corresponding most stable conformers.

### 3.3. Computational Studies

#### 3.3.1. Complete ^1^H NMR Assignment of Cedranolides (2017)

The authors of this publication took as a background the fact that the cedranolid family is made up of sesquiterpene derivatives that share the tricyclo[5.3.1.0^1,5^]undecane skeleton. In this regard, it is important to note that before this publication [42], only incomplete ^1^H NMR data existed for the ten molecules studied.

**Figure 19:**
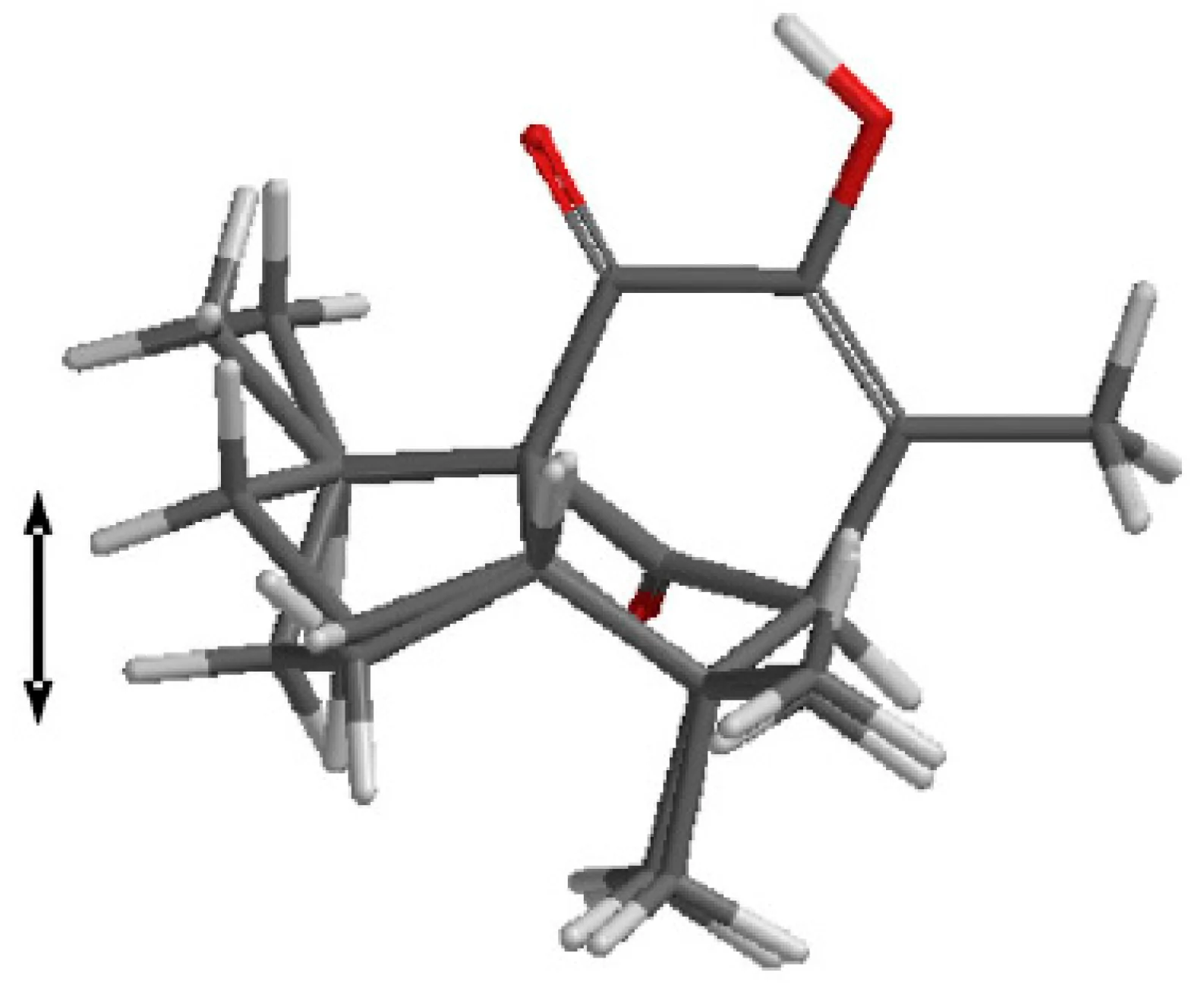
Conformers **1a** and **1b** of α-pipitzol (**3**). Adapted from Ref. [42].

The authors carried out an extensive review of the ^1^H NMR signals of the ten cedranolides evaluated, using the iterative full-spin analysis available in PERCH NMR v.2011.1 software (Perch Solutions Ltd., Kuopio, Finland). In addition, the respective coupling constants were compared with the conformational preferences of the ten cedranolides, highlighting **3**. The work was carried out using density functional theory and a whole-basis-set method (CBS-4M, where M denotes the minimum location of the population) to calculate optimized geometries and then derive Boltzmann distributions (Figure 19).

In addition, it is necessary to keep in mind that this study was carried out at both experimental and theoretical levels, and the respective results were compared and provided feedback based on previous studies.

In particular, interesting information was obtained regarding the target **3** (Table 3a,b). In its ^1^H NMR spectrum, the chemical shifts in the hydrogens of the C10 methyl group (1.382 ppm) on the five-membered ring were slightly higher compared to the rest of the cedranolides. The most notable differences are in the six-membered ring that bears different substituents at C4 (between 1.214 and 2.364 ppm), C5 (from 4.675 to 5.220 ppm), and C6 (between 1.811 and 2.667 ppm). In all cases, the 12-Me hydrogen signal (1.079 ppm) appears slightly downfield of the 13-Me signal (1.029 ppm). From the data in Table 3c, the coupling constants (*J* = 10.2, 8.9, 3.8, 8.2, and 10.2 Hz) of **3** are distinct from of the rest of the studied compounds, since it is the only derivative with a carbonyl group at C4.

Based on conformational analyses, it was possible to separate the compounds into two groups. The first group corresponds to compounds with a rotation of C1-C2-C3-C3a-C8a of the five-membered ring to the conformation of a twisted envelope, with the methyl group at C3 in a pseudo-equatorial orientation. This group includes 1a. In the other group, which includes 1b, the methyl group at C3 adopts a pseudo-axial orientation. It was observed that the most favored conformation for **3** is a twisted form, with the methyl group at C3 in a pseudo-equatorial orientation, with a relative population of 87.4%. This would explain the observed differences between the coupling constant values (Table 4). The six-membered ring of **3** adopts a half-chair conformation.

#### 3.3.2. Vibrational Circular Dichroism Study of Pipitzols and of Their Inverse Epimer Constituents α- and β-Pipitzol (2022)

Experimental VCD spectra were compared with theoretical spectra generated by DFT theory with the B3LYP functional and the DGDZVP basis set. Based on visual inspection, the spectra indicate a good level of agreement [43]. Using CompareVOA software to validate the assignment with a confidence level (C) of 100%, absolute configurations of 3*R*, 3a*R*, 7*R*, and 8a*S* for **3** and 3*R*, 3a*S*, 7*S*, and 8a*R* for **4** were confirmed.

Comparing the experimental spectrum of **3** with the calculated spectrum of **4** in diethyl ether in the ranges 1550–950 cm^−1^ and 1800–950 cm^−1^, enantiomeric similarity indices of −62.0 and −70.9, respectively, were obtained, implying that **3** and **4** are enantiomers rather than inverse epimers. Comparing the experimental spectrum of **4** with the calculated spectrum of **3**, the enantiomeric similarity indices in the same ranges were −47.5 and −63.9, respectively. The first lower index would suggest that they would not be vaguely epimeral, while the second would indicate that they are indeed epimeral.

Finally, the methyl groups of the *gem*-dimethyl arrangements of **3** and **4** were deuterated, in the expectation that this would lead to substantial changes in the spectra. However, this was not the case, since there were no appreciable peaks in the region 1800–950 cm^−1^. In conclusion, VCD seems not to be ideal for distinguishing the inverse epimers of α- and β-pipitzol (**3** and **4**, respectively), with three enantiomeric stereogenic centers and one stereogenic center with the same configuration. Further evaluation of other inverse epimers is necessary to ascertain the limit of distinction of the technique.

#### 3.3.3. Understanding Experimental Facts for the Transformation of Perezone into α- and β-Pipitzols (2024)

**Figure 20:**
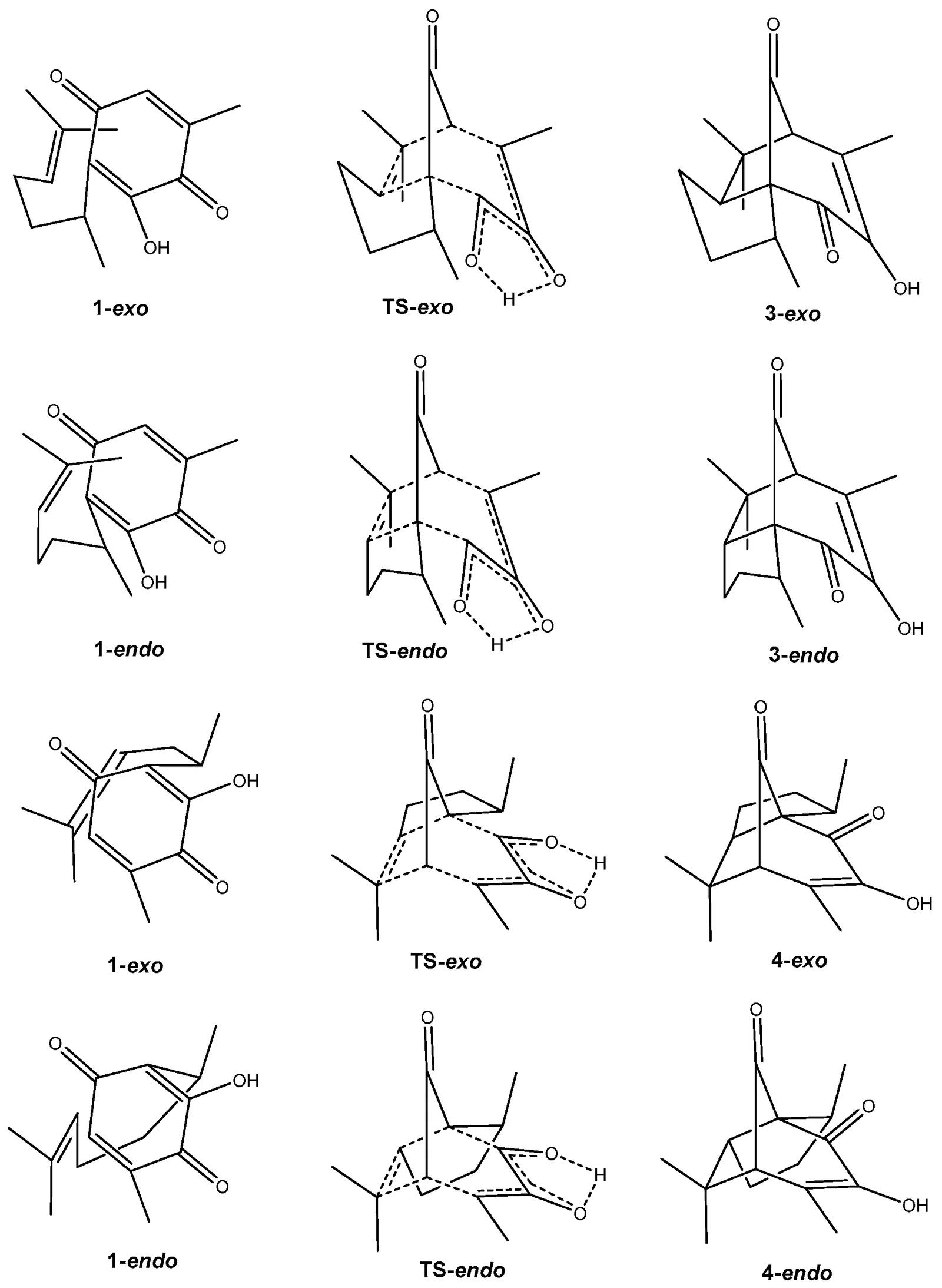
Transition states (TS) of four isomeric pipitzols (**3** and **4**) from **1**. Adapted from Ref. [44].

Murillo-Herrera et al. [44] described the transformation of **1** to **3** and **4**, employing computational calculations at the M06-2x/6-311++G(2d,2p) level of theory. The conformers of **1** display two arrangements of the quinone face related to side-chain approaches, and each of them presents two orientations related to the double bond: *exo* or *endo* (Figure 20). The authors stated that the *endo* adducts display higher angular strain than the *exo* adducts, mainly around C12, resulting in higher steric demand.

Additionally, in the analysis of non-covalent interaction surfaces of transition states (Figure 20) for four adducts, the authors found that the transition state for the **3**-*exo* adduct showed more attractive non-covalent interactions than that for the **3**-*endo* adduct, with a relative difference of 0.04 ua. For repulsive non-covalent interactions, the difference was slightly lower at 0.02 ua. The **4**-*exo* adduct showed less repulsive non-covalent interactions in comparison to the **4**-*endo* adduct, with a difference of 0.06 ua. With regard to attractive non-covalent interactions, both adducts displayed the same value of 0.37 ua.

#### 3.3.4. α, β-Pipitzols and α, β-Isopipitzols from Natural Quinone Perezone: Quantum Chemistry, Docking, Chemoinformatic, and Pharmacological Studies (2026)

Employing the DFT/B3LYP/6-311++G(d,p) method, a theoretical study was conducted to determine the most stable conformers for the title compounds **3**–**6** (Table 5) together with other geometric, chemical, energetic, and spectroscopic (NMR and IR) properties. This study was the first to report the theoretically predicted properties of **3**–**6** [45].

**Figure 21:**
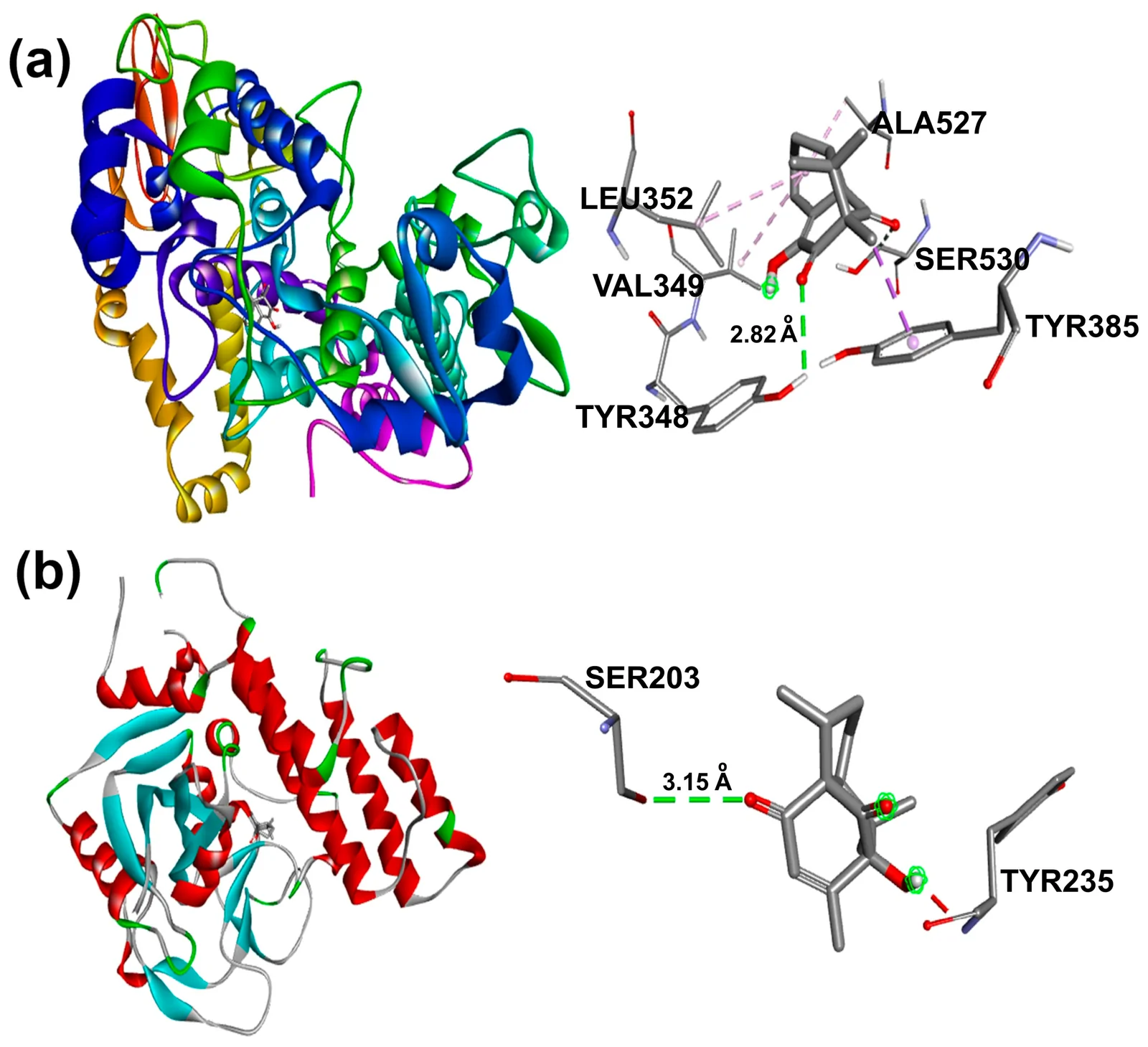
(**a**) Interaction between amino acid residues (Tyr385, Tyr348, and Ser530) of COX-2 and **6**, (**b**) interaction between amino acid residues (Tyr235 and Ser203) of PARP-1 and **6**. Adapted from Ref. [45].

Docking studies were performed using Autodock 4.2 among the enzymes COX-2 (catalytic site Arg120, Tyr385, and Ser530), PARP-1 (catalytic site His201, Tyr235, and Glu327), and molecules **3**–**6** to determine whether any intermolecular interactions arose (Figure 21) [44]. Figure 21 shows, as representative examples, the interactions between COX-2, PARP-1, and **6**. For the enzyme COX-2, there is a hydrogen bond interaction (2.82 Å) between the hydrogen of the hydroxyl group of Tyr348 and the carbonyl group, a π–sigma interaction between the aromatic ring of Tyr385 and the methyl group placed between the two ketone groups, and a carbon–hydrogen interaction with Ser530 (Figure 21a). Concerning the interactions between PARP-1 and **6** (Figure 10b), there is a hydrogen bridge (3.15 Å) between the oxygen of the hydroxyl group of **6** and the hydrogen of the amino group of Ser203. Tyr235 shows an unfavorable interaction with the carbonyl oxygen of **6**.

Compounds **3**, **4**, and **6** were tested in vitro for cytotoxic activity against several tumor cell lines: MDA-MB-231, MCF-7, U373, A549, and U87 (Table 6).

The cell viability percentages obtained indicate that these compounds did not cause mortality at 25 μM in the studied cancer cells [44]. Compound **4** displayed the lowest inhibitory concentration toward the A549 cell line (64.49 µM), while **6** showed the lowest inhibitory concentration of 83.59 µM toward the U373 cell line and 87.85 µM toward the MCF-7 cell line. It is important to note that **1** was studied in relation to the A549 tumor cell line, showing an inhibitory concentration of 143.8 µM, whereas **3**, **4**, and **6** exhibited better inhibitory concentrations. Overall, these compounds are pharmacologically less active than the reference olaparib.

### 3.4. Pharmacological Studies

#### 3.4.1. Antidiabetic and Antihyperalgesic Effects of a Decoction and Compounds from *Acourtia thurberi* (2017)

In this paper [46], the authors begin by highlighting the fact that diabetes is a medical condition that affects millions of people throughout the world, with an expected increase in its prevalence. In this regard, most patients consider naturopathic preparations to be more effective, less toxic, and less expensive than commercial drugs.

Among the different options available, one is widely marketed in contemporary Mexico: *Acourtia thurberi*. Thus, the publication evaluates the efficacy of a decoction of *Acourtia thurberi* (AtD) in treating two types of mice (normal and hyperglycemic). Notably, in experiments conducted according to Lorke’s procedure, at no time were toxic effects observed in the mice.

Oral administration at 316.2 mg/kg in both groups of mice showed a decrease in blood glucose levels, although the effect was smaller than that of glibenclamide (an antidiabetic drug). Given the fact that a dose of 31.6 mg/kg demonstrated a considerable effect, a dose of 10 mg/kg was also tested. The effects were very similar, implying that the action of AtD is not dose-dependent in oral glucose tolerance tests. Subsequently, the potential inhibitory activity of AtD toward α-glucosidase was spectrophotometrically evaluated. The results indicated that the activity of the enzyme was inhibited by 97.5 ± 4.0%, depending on the concentration. Furthermore, local administration at all doses attenuated the duration of neurogenic and anti-inflammatory phases in hyperglycemic and hyperalgesic mice. Similarly, AtD demonstrated antinociceptive and anti-hyperalgesic properties, both centrally and peripherally, in relieving pain in normal and hyperglycemic mice.

To determine the composition of AtD, a UPLC analysis was carried out, which established **1**, **3**, **4**, and 8-β-D-glucopyranosyloxy-4-methoxy-5-methyl-coumarin as the four main components. The identities of these compounds were confirmed by the correlation of their NMR spectra data with the data published in the literature. It is important to note that the four compounds were independent of the abutment evaluated in the same trials as AtD. None of them demonstrated relevant hypoglycemic effects. By performing oral sucrose tolerance tests, it was shown that they are concentration-dependent α-glucosidase inhibitors. Their effects in the anti-inflammatory phase were comparable to that of gabapentin.

#### 3.4.2. α, β-Pipitzols and α, β-Isopipitzols from Natural Quinone Perezone: Quantum Chemistry, Docking, Chemoinformatic, and Pharmacological Studies (2026)

As mentioned in the final paragraph of the previous section, the compounds **3**, **4**, and **6** examined in this study also exhibited lower pharmacological activity against five cancer cell lines (Table 6) [45].

### 3.5. Complementary Information

A set of 47 references [18,36,47,48,49,50,51,52,53,54,55,56,57,58,59,60,61,62,63,64,65,66,67,68,69,70,71,72,73,74,75,76,77,78,79,80,81,82,83,84,85,86,87,88,89,90,91] in which the words pipitzol or isopipitzol are merely mentioned is provided in the Supplementary Information.

### 3.6. Future Perspectives from Our Laboratory

#### Mass Spectrometric Differentiation Between Perezone and Its Four Isomeric Compounds; Plausible, Retro [5+2] and [4+2] Cycloadditions

In our future work, a comprehensive mass spectrometry study will be conducted, employing three ionization modes: electron ionization (EI^+^/70 eV), fast atom bombardment (FAB^+^/6 keV), and direct analysis in real time (DART^+^/19.8 eV), in conjunction with high-resolution data (exact-precise values, error in ppm, elemental composition, and unsaturations). Analysis of the acquired spectra supported by high-resolution data should provide unrivaled insight. Retro cycloadditions may be envisaged: retro [5+2] for α-pipitzol (**3**) and β-pipitzol (**4**); retro [4+2] for β-isopipitzol (**6**). 

The ultimate aim of this work is to provide mass spectrometric fragmentation information to more easily distinguish the five isomeric molecules (**1**–**5**).

## 4. Conclusions

We have presented the first comprehensive and systematic review of the literature on α-pipitzol, β-pipitzol, α-isopipitzol, and β-isopipitzol in the period 1852–2026, with corrected and updated information where appropriate. The collated information should prove valuable for the advancement of this field, being obtained from 148 references cited in specialized databases (ResearchGate, Scopus, and SciFinder^®^).

## Figures and Tables

**Table 1:** References from the main databases.

Keyword	SciFinder^®^	Scopus	ResearchGate
Perezone	118	109	123
Pipitzols	0	13	25
Isopipitzols	0	0	6
α-Pipitzol	24	13	5
β-Pipitzol	23	17	6
α-Isopipitzol	1	2	3
β-Isopipitzol	7	6	3

**Table 2:** Relative abundance percentages of major mass fragments of **3**, **4**, and **6**. Adapted from Ref. [21].

Compound	Fragments									
	M^+^	[M-15]^+^	[M-29]^+^	[M-43]^+^	[M-57]^+^	[M-68]^+^	[M-71]^+^	*m*/*z* 167	*m*/*z* 166	*m*/*z* 138
**3**	100	12	25	39	33	5	18	76	70	3
**4**	100	13	33	51	48	7	27	93	90	18
**6**	61	14	14	11	13	6	16	100	39	29

**Table 3:** (**a**) Hydrogen NMR chemical shifts in the methylcyclopentane portion of **3** (in ppm from TMS). (**b**) Hydrogen NMR chemical shifts in the methylcyclohexane portion and the *gem*-dimethyl signals of **3** (in ppm from TMS). (**c**) Coupling constants of the methylcyclopentane portion of **3**, given in Hertz.

Compound	(a)										
	1*R*	1*S*	2*R*	2*S*	3	Me-10	8a	RMS ^a^			
**3**	1.510	1.769	1.656	1.906	2.408	1.382	2.112	0.099			
	**(b)**										
	**4α**	**4β**	**5α**	**5β**	**6**	**7**	**9α**	**9β**	**Me-11**	**Me-12**	**Me-13**
	-	-	-	-	-	2.836	-	-	2.059	1.079	1.029
	**(c)**										
	**^2^ *J*_1*R*,1*S*_**	**^3^ *J*_1*R*,2*R*_**	**^3^ *J*_1*R*,2*S*_**	**^3^ *J*_1*R*,8a_**	**^3^ *J*_1*S*,2*R*_**	**^3^ *J*_1*S*,2*S*_**	**^3^ *J*_1*S*,8a_**	**^2^ *J*_2*R*,2*S*_**	**^3^ *J*_2*R*,3_**	**^3^ *J*_2*S*,3_**	**^3^ *J*_3,Me-10_**
	−12.52	10.20 11.22 ^b^	5.84 5.76 ^b^	8.94 9.62 ^b^	5.78 5.64 ^b^	3.79 2.43 ^b^	8.21 7.28 ^b^	−12.17	10.25 10.73 ^b^	5.67 4.68 ^b^	7.12

^a^ Root mean square for the comparison of experimental and calculated ^1^H NMR spectra. ^b^ Values in italics are vicinal coupling constants derived from Altona calculations.

**Table 4:** Optimized relative conformer energies ΔG (in kcal/mol) and Boltzmann populations (%) for cedranolides from **3**.

Conformer	ΔG	%
1a	−507,107.15	87.4
1b	1.15	12.6

**Table 5:** Energy optimization of compounds **3**–**6**. Adapted from Ref. [45].

Compound	Energy (Hartree)	Electronic Energy (kcal/mol)	Relative Energy (kcal/mol)
**3**	−809.53	−507,990.37	0
**4**	−809.53	−507,989.47	0.90
**5**	−809.51	−507,975.16	15.21
**6**	−809.51	−507,975.32	15.05

**Table 6:** Inhibitory concentration (IC_50_, μM) of human tumor cell lines for target compounds **3**, **4**, and **6**. Adapted from Ref. [45].

Compound	Tumor Cell Lines				
	MDA-MB-231	MCF-7	U373	A549	U87
**3**	112.62	96.38	92.23	101.45	90.63
**4**	104.36	96.24	87.85	64.49	91.89
**6**	98.23	87.85	83.59	103.21	90.33

## Data Availability

No new data were created or analyzed in this study. Data sharing is not applicable to this article.

## Supplementary Materials

The following supporting information can be downloaded at https://www.mdpi.com/article/10.3390/molecules31183342/s1, Complementary information.
