# Supplementary material for: Pipitzols and Isopipitzols, 1852 to 2026: A Review
Source: Molecules. 2026 Sep 20;31(18):3342. doi: 10.3390/molecules31183342 (PMC13610303; doi:10.3390/molecules31183342)
Supplement: Supplementary file 1 [file molecules-31-03342-s001.zip › molecules-4385399-supplementary.pdf]

Pipitzols and Isopipitzols, 1852 to 2026: A Review

Joel Martínez [1], Cristopher Williams Fuentes Cid [1], Adriana Lizbeth Rivera Espejel [1], María Inés Nicolás-Vázquez [1,*], María Z. Saavedra-Leos [2], Alberto A. Fajardo de la Rosa [3], René Gerardo Escobedo-González [4] and René Miranda Ruvalcaba [1,*]

1 Departamento de Ciencias Químicas, Facultad de Estudios Superiores Cuautitlán Campo 1, Universidad Nacional Autónoma de México, Cuautitlán Izcalli 54740, Estado de México, Mexico; jomtz@comunidad.unam.mx (J.M.); 316048734@cuautitlan.unam.mx (C.W.F.C.); riveraespejeladriana@gmail.com (A.L.R.E.)

2 Coordinación Académica Región Altiplano, Universidad Autónoma de San Luis Potosí, Carretera Cedral km 5+600, Ejido San José de las Trojes Matehuala 78700, San Luis Potosí, Mexico; zenaida.saavedra@uaslp.mx

3 Laboratorio de Ingeniería Química, Facultad de Química, Universidad Nacional Autónoma de México, Alcaldía Coyoacán, Ciudad de México 04510, Mexico; aafdlr@ciencias.unam.mx

4 Department of Industrial Maintenance, Technological University of the City of Juárez, Av. Universidad Tecnológica No. 3051, Col. Lote Bravo II, City of Juárez 32315, Chihuahua, Mexico; rene_escobedo@utcj.edu.mx

* Correspondence: nicovain@comunidad.unam.mx (M.I.N.-V.); mirruv@comunidad.unam.mx (R.M.R.)

CONTENT

Complementary information

Set of 47 references in which the words pipitzols and isopipitzols are only mentioned, no important commentaries are offered.

1. Parker W, Roberts JS, Ramage R. Sesquiterpene biogenesis. Quarterly Rev Chem Soc. 1967;21:331-363. https://doi.org/10.1039/QR9672100331

2. Salazar I, Enriquez R, Diaz E, Walls F. The photochemical rearrangement of 5-(1’,5’-dimethylhex-4’-enyl)-6-methoxy-7a-methyl-3*H*-indazole-4,7(3a*H*,7a*H*)-dione, the pyrazoline derivative of *O*-methylperezone. Austr J Chem. 1974;27:163-169. DOI: https://doi.org/10.1071/CH9740163

3. Ortiz de Montellano B. Empirical aztec medicine. Sci. 1975;188:215-220. DOI: https://doi.org/10.1126/science.1090996

4. Stevens KE, Yates P. Cedrenoid sesquiterpenes. Synthesis of the (±) Stork–Clarke β-diketone. J Chem Soc, Chem Commun. 1980;(21):990-991. DOI: https://doi.org/10.1039/C39800000990

5. Yates P, Stevens KE. Bicyclopentanoid sesquiterpenes. the synthesis of cedranoid sesquiterpenes via the photo-rearrangement of bicyclo[2.2.2]octenones. Tetrahedron. 1981;37:4401-4410. DOI: https://doi.org/10.1016/0040-4020(81)80006-3

6. Joseph-Nathan P, Hernández JD, Roman LU, García GE, Mendoza V. Sesquiterpenes from *Perezia carpholepis*. Phytochemistry. 1982;21:669-672. DOI: https://doi.org/10.1016/0031-9422(82)83162-2

7. Garst ME, McBride BJ, Douglass III JG. Intramolecular cycloadditions with 2-(ω-alkenyl)-5-hydroxy-4-pyrones. Tetrahedron Lett. 1983;24:1675-1678. DOI: https://doi.org/10.1016/S0040-4039(00)81742-6

8. Soriano-Garcia M, Ortiz B, Toscano RA. Structure of 1-hydroxy-5,11,15-trimethylpentacyclo[9.5.1.$0^{2,9}$.$0^{4,8}$.$0^{12,16}$]heptadec-2(9)-en-3-one, $C_{20}H_{28}O_2$. Acta Cryst C. 1984;C40:479-481. DOI: https://doi.org/10.1107/S010827018400456X

9. Bohlmann F, Banerjee S, Jakupovic J, King RM, Robinson H. Bisabolene derivatives and acetylenic compounds from peruvian *Coreopsis* species. Phytochemistry. 1985;24:1295-1297. DOI: https://doi.org/10.1016/S0031-9422(00)81119-X
10. Masami I, Takahiko T, Takeyoshi T. Bitter principles of *Ailanthus altissima* SWINGLE. Conversion of Ailanthone into Shinjulactone C. Bull Chem Soc Japan. 1985;58:2357-2360. DOI: https://doi.org/10.1246/bcsj.58.2357
11. Funk RL, Bolton GL Intramolecular [6+4] and [4+2] tropone-olefin cycloaddition reactions. Facile synthesis of the ingenane ring system. J Am Chem Soc. 1986;108:4655-4657. DOI: https://doi.org/10.1021/ja00275a069
12. Soriano-García M, Walls F, Toscano RA. Structure of (1*S*,3*R*,6*R*,7*S*)-2,2,6,10-Tetramethyl-8,11-dioxotricyclo[5.3.1.$0^{3,7}$]undec-9-en-9-yl acetate (β-pipitzol acetate). Acta Cryst C. 1986;C42:329-331. DOI: https://doi.org/10.1107/S0108270186096294
13. Garst ME, Frazier JD. Reaction of 5-substituted 2-methoxy-1,4-quinones with Boron tribromide. J Org Chem. 1987;52:446-448. DOI: https://doi.org/10.1021/jo00379a026
14. Usubillaga A. Solanudine, a steroidal alkaloid from *Solanum nudum*. Phytochemistry. 1988;27:3031-3032. DOI: https://doi.org/10.1016/0031-9422(88)80726-X
15. Jolad SD, Timmermann BN, Hoffmann JJ, Bates RB, Camou FA, Cole JR. Sesquiterpenoids from *Coreocarpus arizonicus*. Phytochemistry. 1988;27:3545-3548. DOI: https://doi.org/10.1016/0031-9422(88)80765-9
16. Zdero C, Bohlmann F, Sánchez H, Dominguez XA. Isocedrene derivatives and other constituents from *Acourtia nana*. Phytochemistry. 1991;30:2695-2697. DOI: https://doi.org/10.1016/0031-9422(91)85125-j
17. Tsang R, Fraser-Reid B. Pyranose α-enones provided ready access to functionalized trans-decalins via bis-annulated pyranosides obtained by intramolecular Diels-Alder reactions. A key intermediate for forskolin. J Org Chem. 1992;57:1065-1067. DOI: https://doi.org/10.1021/jo00030a005
18. Alonso RA, Vite GD, McDevitt RE, Fraser-Reid B. Radical cyclization routes to bridged pyranosides as precursors of densely functionalized cycloalkanes. J Org Chem. 1992;57:573-584. DOI: https://doi.org/10.1021/jo00028a033
19. Fraser-Reid B, Alonso RA, McDevitt RE, Venkateswara Rao B, Vite GD, Zottola MA. Novel reactions of carbohydrates discovered en route to natural products. Bull Soc Chim Belges. 1992;101:617-626. DOI: https://doi.org/10.1002/bscb.19921010709
20. Rodríguez-Hernández A, Barrios H, Collera O, Enríquez RG, Ortiz B, Sánchez-Obregón R, Walls F, Yuste F, Reynolds WF, Yu M. Isomerization of perezone into isoperezone and preparation of dihydroisoperezinone. Nat Prod Lett. 1994;4:133-139. DOI: https://doi.org/10.1080/10575639408044925
21. Alarcon-Aguilar FJ, Roman-Ramos R, Jimenez-Estrada M, Reyes-Chilpa R, Gonzalez-Paredes B, Flores-Saenz JL. Effects of three mexican medicinal plants (*Asteraceae*) on blood glucose levels in healthy mice and rabbits. J Ethnopharmacol. 1997;55:171-177. DOI: https://doi.org/10.1016/S0378-8741(96)01494-8
22. Grieco PA, Walker JK. Intramolecular cationic [5+2] cycloaddition reactions promoted by trimethylsilyl triflate in 3.0 M lithium perchlorate-ethyl acetate: Application to a formal total synthesis of (±)-isocomene. Tetrahedron. 1997;53:8975-8996. DOI: https://doi.org/10.1016/S0040-4020(97)90404-X
23. Mehta G, Srikrishna A. Synthesis of polyquinane natural products: An update. Chem Rev. 1997;97:671-720. DOI: https://doi.org/10.1021/cr9403650
24. Soriano-García M, Huipe-Nava E, Mendoza González V, Garcia Garibay E. Crystal structure of 1β-hydroxy-β-homopipitzolone (9-hydroxy-2,6,9-trimethyltricyclo[6,3,1,01,6]dodeca-5,10,11,12-tetraone). Anal Sci. 2003;19:801-802. DOI: https://doi.org/10.2116/analsci.19.801
25. Hernández-Carlos B, Burgueño-Tapia E, Joseph-Nathan P. A new coumarin from *Perezia hebeclada*. Magn Reson Chem. 2003;41:962-964. DOI: https://doi.org/10.1002/mrc.1284
26. Chappel D, Russell AT. From α-cedrene to crinipellin B and onward: 25 years of the alkene–arene meta-photocycloaddition reaction in natural product synthesis. Org Biomol Chem. 2006;4:4409-4430. DOI: https://doi.org/10.1039/B614011B
27. Gómez-Serrano G, Cristiani-Urbina E, Villegas-Garrido TL. Time-dependent perezone production in different culture systems of *Acourtia cordata*. Cent Eur J Biol. 2012;7:507-518. DOI:

https://doi.org/10.2478/s11535-012-0035-2
28. Fraga BM. Natural sesquiterpenoids. Nat Prod Rep. 2012;29:1334-1366. DOI: https://doi.org/10.1039/C2NP20074K
29. Presset M, Coquerel Y, Rodriguez J. Syntheses and applications of functionalized bicyclo[3.2.1]octanes: Thirteen years of progress. Chem Rev. 2013;113:525-595. DOI: https://doi.org/10.1021/cr200364p
30. Ylijoki KEO, Stryker JM. [5+2] Cycloaddition reactions in organic and natural product synthesis. Chem Rev. 2013;113:2244-2266. DOI: https://doi.org/10.1021/cr300087g
31. Pellissier H. Stereocontrolled domino reactions. Chem Rev. 2013;113:442-524. DOI: https://doi.org/10.1021/cr300271k
32. Zepeda LG, Burgueño-Tapia E, Perez-Hernandez N, Cuevas G, Joseph-Nathan P. NMR-based conformational analysis of perezone and analogues. Magn Reson Chem. 2013;51:245-250. DOI: https://doi.org/10.1002/mrc.3940
33. Ramírez-Gualito K, López-Mora N, Jiménez-Vázquez HA, Tamariz J, Cuevas G. The role of supramolecular intermediates in the potential energy surface of the Diels-Alder reaction. J Mex Chem Soc. 2013;57:267-275. DOI: https://www.scielo.org.mx/pdf/jmcs/v57n4/v57n4a2.pdf
34. Clavier H, Pellissier H. Recent developments in the [5+2] cycloaddition. Methods and Applications of Cycloaddition Reactions in Organic Syntheses. Chapter 20. pp. 631-654, 2014. DOI: https://doi.org/10.1002/9781118778173.ch20
35. Georgantea P, Ioannou E, Vagias C, Roussis V. Bisabolane and chamigrane sesquiterpenes from the soft coral *Pseudopterogorgia rigida*. Phytochem Lett. 2014;8:86-91. DOI: https://doi.org/10.1016/j.phytol.2014.02.006
36. Sharma MK, Banwell MG, Willis AC. Generation of (+)-prezizanol, (+)-prezizaene, and the *ent*-β-isopipitzol framework via cationic rearrangement of Khusiol and related compounds. Asian J Org Chem. 2014;3:632-637. DOI: https://doi.org/10.1002/ajoc.201400019
37. Reyes-López E, Quiroz-García B, Carpio-Martínez P, Jiménez-Barbero J, Cortés-Guzmán F, Esturau-Escofet N, Cuevas G. The folded conformation of perezone revisited. Long range nOe interaction in small molecules: Interpretable small signals or useless large artifacts? J Mex Chem Soc. 2017;61:177-185. DOI: https://www.scielo.org.mx/pdf/jmcs/v61n3/1870-249X-jmcs-61-03-00177.pdf
38. Le Bideau F, Kousara M, Chen L, Wei L, Dumas F. Tricyclic sesquiterpenes from marine origin. Chem Rev. 2017;117:6110-6159. DOI: https://doi.org/10.1021/acs.chemrev.6b00502
39. Luna-Vázquez FJ, Ibarra-Alvarado C, Camacho-Corona MR, Rojas-Molina A, Rojas-Molina JI, García A, Bah M. Vasodilator activity of compounds isolated from plants used in mexican traditional medicine. Molecules. 2018;23:1-17. DOI: https://doi.org/10.3390/molecules23061474
40. Gao K, Zhang Y, Wang Z, Ding H. Recent development on the [5+2] cycloadditions and their application in natural product synthesis. Chem Commun. 2019;55:1859-1878. DOI: https://doi.org/10.1039/C8CC09077G
41. Armenta-Salinas C, Guzmán-Mejía R, García-Gutiérrez HA, Román-Marín LU, Hernández-Hernández JD, Cerda-García-Rojas CM, Joseph-Nathan P. Novel sesquiterpene skeletons by multiple Wagner–Meerwein rearrangements of a longipinane-1,9-diol derivative. J Nat Prod. 2019;82:3410-3420. DOI: https://doi.org/10.1021/acs.jnatprod.9b00784
42. Escandón-Rivera SM, Mata R, Andrade-Cetto A. Molecules isolated from mexican hypoglycemic plants: A review. Molecules. 2020;25:1-33. DOI: https://doi.org/10.3390/molecules25184145
43. Gao K, Hu J, Ding H. Tetracyclic diterpenoid synthesis facilitated by ODI-cascade approaches to bicyclo[3.2.1]octane skeletons. Acc Chem Res. 2021;54:875-889. DOI: https://doi.org/10.1021/acs.accounts.0c00798
44. George JH. Biomimetic dearomatization strategies in the total synthesis of meroterpenoid natural products. Acc Chem Res. 2021;54:1843-1855. DOI: https://doi.org/10.1021/acs.accounts.1c00019
45. Quiñonez-Bastidas GN, Navarrete A. Mexican plants and derivates compounds as alternative for inflammatory and neuropathic pain treatment—A review. Plants. 2021;10:1-30. DOI: https://doi.org/10.3390/plants10050865
46. Hernández-Pérez A, Campos-Montiel RG, López-Palestina CU, Juárez-Maldonado A,

Hernández-Fuentes AD. Medicinal plants of the *Asteraceae* family with hypoglycemic activity in Mexico. Boletín De Ciencias Agropecuarias Del ICAP. 2022;8:14-17. DOI: https://doi.org/10.29057/icap.v8i16.8274
47. Yang L, Zhang C, Sun J. Catalytic enantioselective perezone-type [5+2] cycloaddition. J Am Chem Soc. 2025;147:39860-39869. DOI: https://doi.org/10.1021/jacs.5c14484
